# Optodynamic simulation of β-adrenergic receptor signalling

**DOI:** 10.1038/ncomms9480

**Published:** 2015-09-28

**Authors:** Edward R. Siuda, Jordan G. McCall, Ream Al-Hasani, Gunchul Shin, Sung Il Park, Martin J. Schmidt, Sonya L. Anderson, William J. Planer, John A. Rogers, Michael R. Bruchas

**Affiliations:** 1 Department of Anesthesiology, Division of Basic Research, Washington University School of Medicine, St Louis, Missouri 63110, USA; 2 Washington University Pain Center, Washington University School of Medicine, St Louis, Missouri 63110, USA; 3 Division of Biology and Biomedical Sciences, Washington University School of Medicine, St Louis, Missouri 63110, USA; 4 Anatomy and Neurobiology, Washington University School of Medicine, St Louis, Missouri 63110, USA; 5 Department of Materials Science and Engineering, Frederick Seitz Materials Research Laboratory, University of Illinois at Urbana-Champaign, Urbana, Illinois 61801, USA; 6 Department of Electrical and Computer Engineering, University of Illinois at Urbana-Champaign, Urbana, Illinois 61801, USA; 7 Department of Mechanical Science and Engineering, University of Illinois at Urbana-Champaign, Urbana, Illinois 61801, USA; 8 Department of Chemistry, University of Illinois at Urbana-Champaign, Urbana, Illinois 61801, USA; 9 Department of Biomedical Engineering, Washington University in St Louis, St Louis, Missouri 63110, USA

## Abstract

Optogenetics has provided a revolutionary approach to dissecting biological phenomena. However, the generation and use of optically active GPCRs in these contexts is limited and it is unclear how well an opsin-chimera GPCR might mimic endogenous receptor activity. Here we show that a chimeric rhodopsin/β_2_ adrenergic receptor (opto-β_2_AR) is similar in dynamics to endogenous β_2_AR in terms of: cAMP generation, MAP kinase activation and receptor internalization. In addition, we develop and characterize a novel toolset of optically active, functionally selective GPCRs that can bias intracellular signalling cascades towards either G-protein or arrestin-mediated cAMP and MAP kinase pathways. Finally, we show how photoactivation of opto-β_2_AR *in vivo* modulates neuronal activity and induces anxiety-like behavioural states in both fiber-tethered and wireless, freely moving animals when expressed in brain regions known to contain β_2_ARs. These new GPCR approaches enhance the utility of optogenetics and allow for discrete spatiotemporal control of GPCR signalling *in vitro* and *in vivo*.

## Abstract (web summary)

Optogenetic activation of β2-adrenergic receptors (β2-AR) has been achieved, but not characterized in detail. Here, Siuda *et al*. show that light-controlled opto-β2AR mimics endogenous β2AR activity *in vitro* and *in vivo*, and develop novel, optically active, functionally selective receptors to bias β2AR intracellular signaling mechanisms.

## Graphical abstract

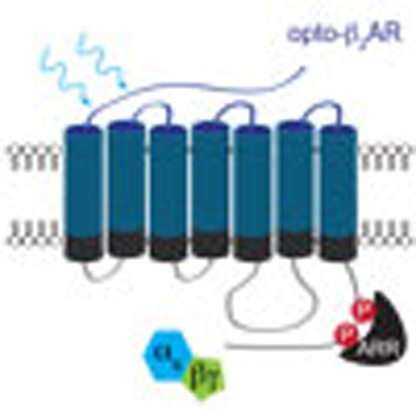

Over the past decade, optogenetics and chemogenetics have made significant contributions to probing biological questions. Traditional optogenetic approaches however, utilize expression of depolarizing (channelrhodopsins)^1^,^2^,^3^ and hyperpolarizing (halorhodopsins and archaerhodopsins)^4^ ion channels to selectively turn on/off neurons in the presence of light. Chemogenetic approaches, such as designer receptors exclusively activated by designer drugs^5^, have high utility for their modulation of GPCR signalling, but similar to other ligand-mediated responses, can sometimes be limited by pharmacokinetics and pharmacodynamics making them difficult to use to examine real-time kinetics of receptor activity, particularly *in vivo.* Thus, the development of optically active G-protein coupled receptors (GPCRs) allows for more fine tuned modulation of cellular activity. Through manipulation of the light stimulus, we can modulate the activity of optically active GPCRs in an ‘optodynamic’ manner. This, in combination with the spatiotemporal control offered through optogenetics, provides a more refined *in vivo* GPCR toolkit than is currently possible with chemogenetics and traditional pharmacology.

The chimeric rhodopsin/β_2_ adrenergic receptor (opto-β_2_AR) has been shown to activate cAMP, presumably through Gαs-mediated signalling, and to modulate neuronal excitability^6^,^7^,^8^. However, it is unclear whether opto-β_2_AR behaves similarly to endogenous β_2_AR. Here we fully evaluated the *in vitro* activity of opto-β_2_AR and β_2_AR by examining the temporal kinetics of cAMP and MAP kinase activation in addition to receptor internalization and desensitization, and demonstrate that opto-β_2_AR mimics the dynamic signalling profile of β_2_ARs.

Over the past few years the tenets of GPCR pharmacology have been challenged by the concept of ‘functional selectivity’ or ‘biased agonism,’ demonstrating that ligands exert varying levels of efficacies on intracellular signalling mechanisms and that G-proteins may not be the sole determinants of intracellular activity^9^,^10^,^11^. While arrestin was canonically thought to only terminate GPCR-mediated signalling through inactivation and internalization of the receptor, it is now widely accepted that arrestin acts to scaffold several intracellular signalling cascades, particularly MAP kinases. Several receptors, including opioid receptors^12^,^13^,^14^, angiotensin II^15^, V2 vasopressin^16^ and βAR^17^ display arrestin-dependent MAP kinase activation. In an effort to combine spatiotemporal control of opto-β_2_AR with biased GPCR signalling, we performed site-directed mutagenesis on opto-β_2_AR to generate optically active, functionally selective GPCRs.

Mutation of three key residues in β_2_AR (β_2_AR^T68F,Y132G,Y219A^ or β_2_AR^TYY^) generate an arrestin-biased mutant^11^, while modification of C-terminal serines prevents arrestin binding by blocking G-protein-coupled receptor kinase phosphorylation resulting in a G-protein-biased mutant (β_2_AR^S355A,S356G^ or β_2_AR^SS^)^18^,^19^,^20^,^21^. Analogous residues were altered in opto-β_2_AR to generate an arrestin-biased receptor, opto-β_2_AR^L72F,Y136G,Y224A^ or opto-β_2_AR^LYY^ and a G-protein-biased receptor, opto-β_2_AR^S362A,S363G^ or opto-β_2_AR^SS^. Here we determined the dynamic optical properties of these novel optically active, functionally selective receptors.

Heterologous expression systems are essential in characterization of receptor activity. They offer the ability to dissect receptor function not possible in more complex environments. However, to truly understand endogenous activity, it is essential to ultimately look at *in vivo* function. To that end, we determined whether opto-β_2_AR could be used *in vivo* since it has yet to be utilized for inducing a significant behavioural phenotype^6^. We expressed opto-β_2_AR in a biologically relevant neural circuit known to be under the influence of noradrenergic signalling and that expresses β_2_ARs and its signalling moieties, the basolateral amygdala (BLA). The presence of all nine adrenergic receptor subtypes within the amygdaloid complex^22^ has precluded determination of their roles and signalling pathways *in vivo* due to the fact that pharmacological isolation of these receptors is difficult within the amygdala. These caveats are true for many GPCRs, due to the lack of spatiotemporal control of receptor function, isolation of specific cell types, and control of select noradrenergic or other modulatory inputs. To isolate noradrenergic GPCR signalling *in vivo*, we used opto-β_2_AR and demonstrated that *in vivo* photoactivation of β-adrenergic signalling produced excitation of BLA neurons resulting in anxiety-like states in both fiber-tethered and wireless, freely moving animals.

Here we have fully evaluated the utility of opto-β_2_AR in mimicking endogenous β_2_AR activity; we developed novel, optically active, functionally selective receptors to bias β_2_AR intracellular signalling mechanisms and we used opto-β_2_AR *in vivo* and define its ability to initiate a series of real-time behavioural responses.

## Results

### Optical control of β-adrenergic signalling

**Figure 1:**
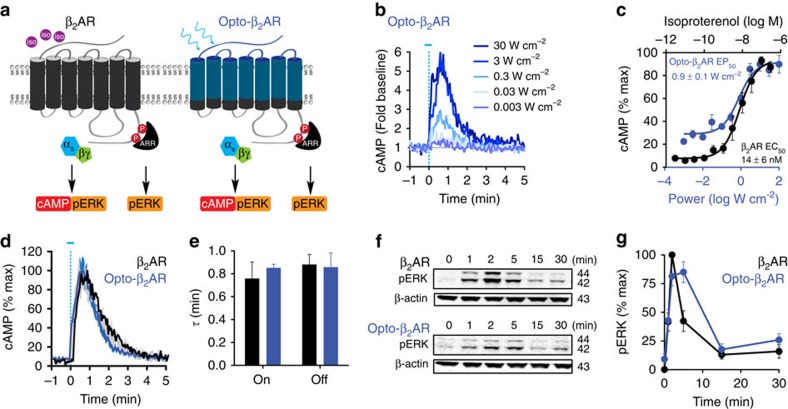
Opto-β_2_AR and β_2_AR exhibit similar G-protein signaling mechanisms. (**a**) Both β_2_AR (ligand) and opto-β_2_AR (light) activate intracellular cAMP and pERK pathways. (**b**) Representative traces show light-induced activation of cAMP in response to increasing powers of light (5 s pulse) in HEK293 cells expressing opto-β_2_AR. (**c**) Power response curve of cAMP of opto-β_2_AR (blue) (EP_50_=0.9±0.1 W cm^−2^; *n*=4 experiments). Isoproterenol increase cAMP in β_2_AR (black) expressing cells (EC_50_=14±6 nM; *n*=6 experiments). (**d**) Opto-β_2_AR (blue, *n*=14 experiments) and endogenous β_2_AR in HEK293 cells (black, *n*=4 experiments) display similar kinetics of cAMP activation and deactivation in response to photostimulation (5 s pulse) and isoproterenol (1 μM) respectively (mean=solid line, s.e.m.=shaded area). (**e**) Time constants of cAMP activation (*τ*_on_) and deactivation (*τ*_off_) fit from traces in **d** for opto-β_2_AR (blue) and β_2_AR (black). Activation (opto-β_2_AR=0.86±0.12 min; *n*=18 experiments and β_2_AR=0.77±0.14 min; *n*=4 experiments) and deactivation (opto-β_2_AR=0.85±0.03 min; *n*=15 experiments and β_2_AR=0.88±0.09 min; *n*=4 experiments) time constants are not statistically different. (**f**) Representative pERK immunoblots in response to isoproterenol (1 μM) in β_2_AR and photostimulation (1 min) in opto-β_2_AR. (**g**) Quantification of immunoblots for both β_2_AR (black, *n*=5 experiments) and opto-β_2_AR (blue, *n*=8 experiments) displayed over time. All data are expressed as mean±s.e.m. All light pulses are 473 nm, 1 W cm^−2^ unless otherwise noted.

We first fully characterized a unique optical tool for activating β-adrenergic signalling and compared its pharmacodynamic properties with β_2_-adrenergic receptors (β_2_AR). The opto-β_2_AR receptor is a chimeric protein that includes transmembrane and extracellular components of bovine rhodopsin, with intracellular domains and loops of the β_2_ adrenergic receptor (Fig. 1a)^6^. Photostimulation of HEK293 cells expressing opto-β_2_AR caused a real-time, light-power-dependent increase in cAMP (cyclic adenosine monophosphate), a canonical product of the Gαs signalling pathway (Fig. 1b,c), similar to isoproterenol-induced concentration-dependent cAMP generation (Fig. 1c)^23^. This real-time cAMP increase in response to light was absent in untransfected HEK293 cells (Supplementary Information, Supplementary Fig. 1a). Furthermore, the kinetics of cAMP activation (*τ*_on_) and inactivation (*τ*_off_) are strikingly similar between opto-β_2_AR and endogenous β_2_AR in HEK293 cells suggesting that although the extracellular regions of the receptors differ greatly, the conformational change required to initiate intracellular signalling are maintained, making these receptors kinetically similar (Fig. 1d,e). In addition, cAMP triggers activation of cyclic nucleotide-gated nonspecific cation channels. Here we show opto-β_2_AR causes a robust increase in intracellular Ca^+2^ in response to light stimulation, with similar results obtained for β_2_AR following isoproterenol bath application, while untransfected HEK293 cells show no response to light (Supplementary Fig. 1b).

In addition to cAMP, extracellular-signal regulated kinase (ERK) 1/2 phosphorylation has been examined extensively in β_2_AR, showing a rapid, yet transient peak within 2–5 min of isoproterenol-induced activation^11^,^17^,^24^,^25^,^26^. To determine whether opto-β_2_AR also activates ERK 1/2 kinases, we stimulated opto-β_2_AR with a 1 min light pulse and generated a time course of ERK phosphorylation (pERK). Similar to isoproterenol-induced pERK in β_2_AR, opto-β_2_AR showed a rapid and transient increase in pERK that peaks within 2–5 min and then rapidly declines (Fig. 1f,g, Supplementary Fig. 2a for full time course). The kinetic effects seen in β_2_AR were the same whether in the continued presence of isoproterenol (Fig. 1f,g) or following a 1 min pulse with isoproterenol (Supplementary Fig. 2b,c). We also show that levels of total ERK in β_2_AR remain constant over a two-hour trial period, suggesting the kinetics of pERK are not due to degradation of total ERK (Supplementary Fig. 2d,e). In addition, the extent of ERK activation in opto-β_2_AR displayed a light-power-dependent relationship that was absent in untransfected HEK293 control cells (Supplementary Fig. 2f,g). The frequency of the light pulse showed the most effect on pERK at full (not pulsed) light or at 5 s on/5 s off (Supplementary Fig. 2h), while light pulse length had little effect (Supplementary Fig. 2i), suggesting that opto-β_2_AR activity can be modulated via manipulation of the light stimulus (that is, optodynamic). These kinetically parallel data sets suggest that photoactivation of opto-β_2_AR induces rapid and transient increases in receptor signalling known to be mediated through β-adrenergic, Gαs-dependent pathways^11^.

Previous studies have shown that rhodopsin and some chimeric GPCRs display dark activity, or are constitutively active in the absence of light^7^,^27^,^28^,^29^. To test this, we quantified levels of pERK in HEK293 cells stably expressing opto-β_2_AR and untransfected controls. In the absence of light stimulation, both cell types showed similar levels of pERK, suggesting that the presence of opto-β_2_AR does not induce constitutive activity (Supplementary Fig. 2j)^6^,^30^,^31^.

### Opto-β_2_AR internalization and desensitization

**Figure 2:**
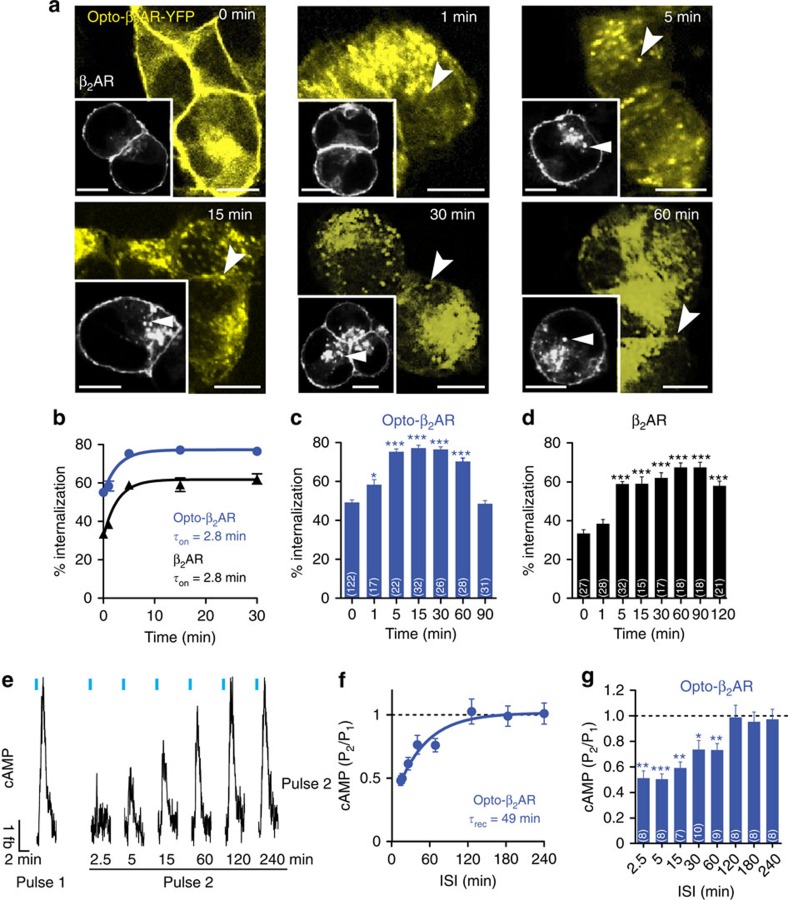
Opto-β_2_AR and β_2_AR internalize and recover from desensitization. (**a**) Representative images show internalization of opto-β_2_AR–YFP in response to photostimulation (1-min pulse). Inset shows similar internalization of β_2_AR–YFP (colourized to black and white) in response to isoproterenol (1 μM) at: 1, 5, 15, 30 and 60 min post-stimulation. Scale bar,10 μm. Arrowheads show examples of internalized punctate receptors. (**b**) Quantification of internalization in opto-β_2_AR (blue; *τ*_on_=2.8 min) and β_2_AR (black; *τ*_on_=2.8 min) with similar time constants of activation (*τ*_on_). (**c**) Percent internalization for opto-β_2_AR–YFP (*P<0.05, ***P<0.001 via one-way ANOVA followed by Dunnett’s multiple comparison test to 0-min control; (*n*=number of cells per time point). (**d**) Percent internalization in β_2_AR–YFP (***P<0.001 via one-way ANOVA followed by Dunnett’s multiple comparison test to 0 min control; (*n*=number of cells per time point). (**e**) Representative traces of recovery from desensitization in opto-β_2_AR (P1 and P2; 5 s). (**f**) P2/P1 quantification of Fig. 1e, *τ*_rec_=49 min (*n*=6–9 experiments per time point). (**g**) P2/P1 opto-β_2_AR functional recovery (*P<0.05, **P<0.01, ***P<0.001 via paired Student’s paired, two-tailed *t*-tests comparing P2 to P1 at each time point; (*n*=number of experiments). All data are expressed as mean±s.e.m. All light pulses are 473 nm, 1 W cm^−2^ unless otherwise noted.

We also determined if opto-β_2_ARs are regulated through their receptor internalization and desensitization kinetics in a manner similar to β-adrenergic receptors^32^,^33^. Photostimulation (1 min) of opto-β_2_AR resulted in rapid receptor internalization within 2–5 min following light exposure (*τ*_on_=2.8 min) that peaked within 15 min and returned to baseline levels 90 min later (Fig. 2a–c, Supplementary Fig. 3a). In the continued presence of isoproterenol (1 μM), β_2_AR internalization was temporally matched (*τ*_on_=2.8 min) to opto-β_2_AR and yielded values similar to those obtained by other groups (Fig. 2a–d, Supplementary Fig. 3a,b)^23^,^34^. To better mimic the optodynamic stimulation of opto-β_2_AR, β_2_AR cells were treated with a 1 min isoproterenol pulse. β_2_ARs internalized with similar kinetics (*τ*_on_=2.2 min) as opto-β_2_AR, yet in contrast to continuous agonist exposure, β_2_ARs return to baseline more rapidly following a 1-min pulse of agonist (Supplementary Fig. 3c,d). If we compare Fig. 2d and Supplementary Fig. 3d, there are significant differences at the 60, 90 and 120 min time points suggesting that β_2_ARs return faster to the membrane in the absence of agonist, than in its presence (Supplementary Fig. 3e). These kinetic differences in receptor internalization and recycling highlight a significant limitation of traditional pharmacological approaches, as it can be difficult to rapidly remove ligand from the cell media/bath, or in particular following *in vivo* infusion. This dynamic function of the optically active GPCR, highlights the utility of these types of optical approaches that mimic GPCR activity at time scales matched to endogenous neuromodulator (NE) uptake and degradation^35^,^36^.

We next determined the functional recovery from desensitization of opto-β_2_AR in a real-time cAMP assay. Following an initial light pulse (P1), cells produced less cAMP in response to a second light pulse (P2) at short interstimulus intervals (ISI; Fig. 2e). Varying ISIs showed complete functional recovery of cAMP over time (*τ*_rec_=49 min) (Fig. 2e–g). We also observed that the reduced cAMP responses seen at short ISIs are not due to degradation of the 9-*cis* chromophore, but rather internalization and desensitization of the receptor (Supplementary Fig. 3f)^7^. These results suggest that opto-β_2_AR has optodynamically matched kinetics and signal transduction profiles to β_2_AR and is a useful tool for spatiotemporal control of β-adrenergic signalling.

### Functionally selective opto-β_2_ARs receptors

Rhodopsin and βARs are both Class A GPCRs and hence share similar sequence homology (Supplementary Fig. 4). It is this high homology that facilitated the generation of chimeric opto-β_2_ARs to mimic βARs intracellular signalling. In an effort to combine the spatiotemporal control of opto-β_2_AR with biased intracellular signalling cascades, we performed site-directed mutagenesis on opto-β_2_AR to generate optically active, functionally selective, G-protein-coupled receptors.

**Figure 3:**
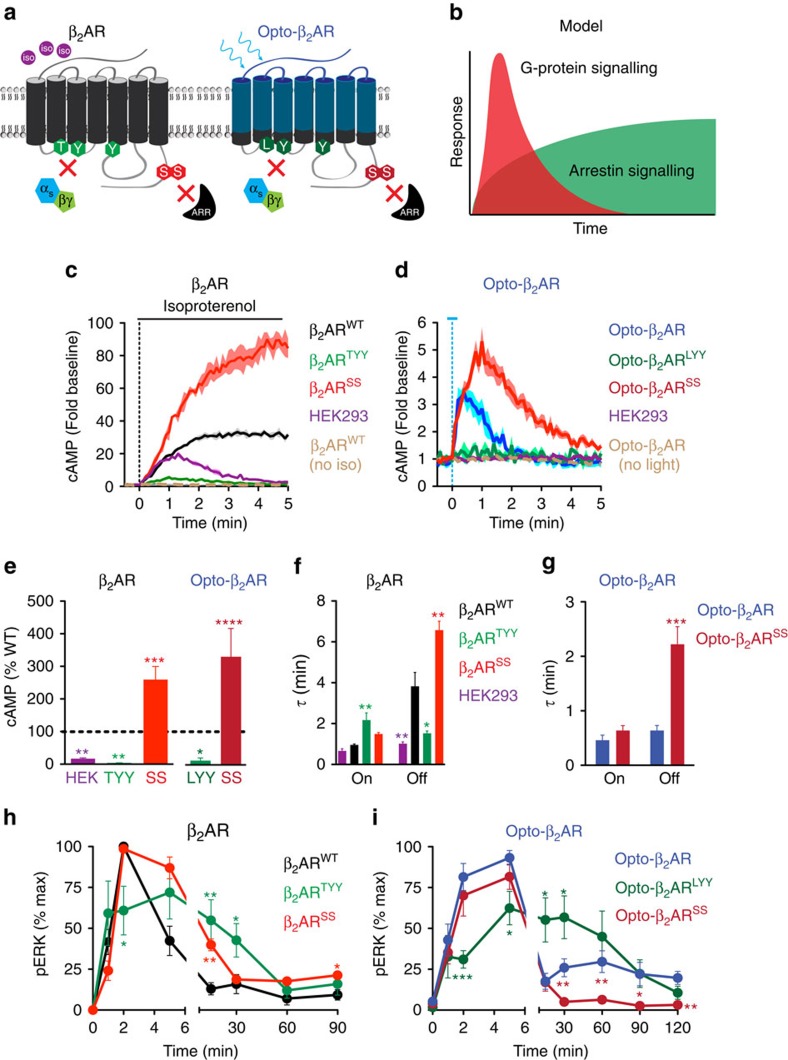
Mutations of β_2_AR and opto-β_2_AR alter intracellular signaling kinetics. (**a**) Schematic of point mutations. (**b**) Model of G-protein and arrestin / MAP kinase signaling^10^. (**c**) Isoproterenol-induced (1 μM) cAMP kinetics of β_2_AR^WT^ (black; *n*=3), β_2_AR^TYY^ (green; *n*=3), β_2_AR^SS^ (red; *n*=3) and HEK293 cells (purple; *n*=3). β_2_AR^WT^ with no isoproterenol (dashed brown; *n*=3; mean=solid line, s.e.m.=shaded area). (**d**) Light-induced (5 s) cAMP kinetics of opto-β_2_AR (dark blue; *n*=8), opto-β_2_AR^LYY^ (dark green; *n*=5) and opto-β_2_AR^SS^ (dark red; *n*=5). HEK293 controls and opto-β_2_AR with no light (dashed brown; *n*=3) (mean=solid line, s.e.m.=shaded area). (**e**) β_2_AR^TYY^ (green; *n*=3), opto-β_2_AR^LYY^ (dark green; *n*=5), HEK293 cells (purple; *n*=3), β_2_AR^SS^ (red; *n*=3) and opto-β_2_AR^SS^ (dark red; *n*=5) cAMP compared with WT (*P<0.05, **P<0.01, ***P<0.001, ****P<0.0001 via one-way ANOVA followed by Dunnett’s multiple comparison test to WT). (**f**) Time constants of isoproterenol (1 μM)-induced cAMP of β_2_AR^WT^ (black; *n*=3), β_2_AR^TYY^ (green; *n*=3), β_2_AR^SS^ (red; *n*=3) and HEK293 cells (purple; *n*=3) (*P<0.05, **P<0.01 via One Way ANOVA followed by Dunnett’s multiple comparison test to WT). (**g**) Time constants of light (5 s pulse)-induced cAMP activation of opto-β_2_AR (dark blue; *n*=8) and opto-β_2_AR^SS^ (dark red; *n*=5) (***P<0.001 via Student’s unpaired, two-tailed *t*-tests to WT). (**h**) Time course of isoproterenol (1 μM)-induced pERK in β_2_AR^WT^ (black; *n*=5), β_2_AR^TYY^ (green; *n*=4) and β_2_AR^SS^ (red; *n*=3) (*P<0.05, **P<0.01 via Student’s unpaired, two-tailed *t*-tests to WT). (**i**) Time course of light (1 min)-induced pERK in opto-β_2_AR (dark blue; *n*=9), opto-β_2_AR^LYY^ (green; *n*=8) and opto-β_2_AR^SS^ (red; *n*=9; *P<0.05, **P<0.01, ***P<0.001 via Student’s unpaired, two-tailed *t*-tests to WT). All data expressed as mean±s.e.m.

Opto-β_2_AR was altered to generate the putative arrestin-biased, opto-β_2_AR^L72F,Y136G,Y224A^ or opto-β_2_AR^LYY^, and the putative G-protein-biased, opto-β_2_AR^S362A,S363G^ or opto-β_2_AR^SS^ (Fig. 3a, Supplementary Fig. 4). It has been proposed and demonstrated by several groups that G-protein-mediated signalling is rapid and transient while arrestin-mediated signalling is slow and prolonged^10^,^11^,^17^ (Fig. 3b). Here we show that activation of endogenous βAR in HEK293 cells with isoproterenol shows a rapid and transient increase in cAMP (Fig. 3c). In contrast, HEK293 cells overexpressing the arrestin-biased, β_2_AR^TYY^ shows a marked reduction in cAMP. This reduction is suggestive of a decrease in G-protein interaction, and may also indicate a potential dominant negative effect of the mutant receptor on endogenous βAR. In contrast, HEK293 cells overexpressing the G-protein-biased β_2_AR^SS^ had an exaggerated response in the continued presence of isoproterenol (Fig. 3c). Interestingly, HEK293 cells overexpressing β_2_AR^WT^ show a longer and sustained cAMP response in the presence of isoproterenol as compared to endogenous βAR in HEK293 cells. These kinetic differences are also clearly seen when repeated at 25 °C (Supplementary Fig. 5a). Further, all three receptor types show similar kinetic responses to forskolin, a general activator of adenylate cyclase (Supplementary Fig. 5b), suggesting these kinetic differences are mediated through Gαs signalling and not due to cAMP sensor expression.

In response to a 5-s pulse of blue light, opto-β_2_AR shows a rapid and transient increase in cAMP, while the arrestin-biased, opto-β_2_AR^LYY^, shows an attenuated response to photostimulation (Fig. 3d). When we repeat this experiment at 25 °C, opto-β_2_AR^LYY^ does show a small response (Supplementary Fig. 5c), yet is still significantly reduced from opto-β_2_AR, similar to the differences we and others have seen with β_2_AR^TYY^ following agonist treatment^11^. We next used an inhibitor of the Gαs subunit (NF 449). Using a concentration shown to effectively reduce isoproterenol-induced cAMP in β_2_AR^WT^ cells (Supplementary Fig. 6a), we show that NF 449 (100 μM) reduces peak cAMP (Supplementary Fig. 6b,d). However, due to the potential for off target activity at purinergic receptors, we used a nonselective P2 purinergic antagonist, suramin (100 μM), and showed no reduction in peak cAMP (Supplementary Fig. 6c,d), suggesting the effect is not due to purinergic receptor interaction but likely to Gαs inhibition. This reduction in real-time cAMP activity is similar to the profile of opto-β_2_AR^LYY^ (Supplementary Fig. 5c), and suggests that the reduction in cAMP is likely due to reduced association between the Gαs subunit and the receptor.

In contrast, the G-protein-biased, opto-β_2_AR^SS^ yields an exaggerated and prolonged cAMP response following photostimulation (Fig. 3d); an effect that is also reproduced at 25 °C (Supplementary Fig. 5c). Comparatively, β_2_AR^SS^ and opto-β_2_AR^SS^ show a significant robust enhancement of cAMP responses when compared to WT, whereas both β_2_AR^TYY^ and opto-β_2_AR^LYY^ show a significant reduction (Fig. 3e).

The differences seen between receptors in activation (*τ*_on_) and deactivation (*τ*_off_) time constants attest to the unique kinetics of each receptor type at both 37 °C (Fig. 3f) and 25 °C (Supplementary Fig. 5d). These kinetic differences may be amplified at cooler temperatures due to a reduction of cellular metabolism, and/or variations in the temperature sensitivity of the ligand-induced conformation versus photoisomerization of retinal. However, we did identify significant differences in the deactivation time constants (*τ*_off_) for both β_2_AR^SS^ and opto-β_2_AR^SS^, which were significantly slower when compared with β_2_AR^WT^ and opto-β_2_AR, respectively (Fig. 3f,g, Supplementary Fig. 5d). These slower rates suggest a lack of G-protein coupled receptor kinase and arrestin recruitment to the membrane prolonging the activity of the receptor and yielding more cAMP output. In contrast, the reduced cAMP levels produced by both β_2_AR^TYY^ and opto-β_2_AR^LYY^ is potentially due to inefficient coupling to the G-proteins that are required to initiate cAMP production. It is also unlikely that these kinetic differences are due to different light transduction properties of each receptor type as all three opto-β_2_ARs light power response curves yield similar EP_50_ values (Supplementary Fig. 5e,f), although we did note that the efficacy for generation of cAMP by opto-β_2_AR is not completely recapitulated comparedwith β_2_AR. This is most likely due to the presence of endogenous β-adrenergic receptors expressed in HEK293 cells.

To confirm that the kinetic cAMP differences observed between opto-β_2_AR, opto-β_2_AR^LYY^ and opto-β_2_AR^SS^ are due to biased intracellular signalling and not receptor expression levels, we quantified cell surface receptor expression. Using on-cell westerns^37^,^38^ with a rhodopsin antibody, we show that rhodopsin expression is not only significantly elevated in the three cell lines in comparison to untransfected HEK293, but are also equal (Supplementary Fig. 5g,h). We also calculated receptor surface expression as a percent of total fluorescence and show that both opto-β_2_AR^LYY^ and opto-β_2_AR^SS^ have increased surface expression compared to opto-β_2_AR (Supplementary Fig. 5i). The reduction in opto-β_2_AR surface fluorescence is most likely due to the higher levels of diffuse internalized receptor at baseline (see 0-min time point in Fig. 2b,c).

Activation of MAP kinase cascades also shows dramatic kinetic differences reminiscent of the model proposed by Luttrell and Getsey–Palmer (Fig. 3b)^10^. In the presence of isoproterenol, β_2_AR^WT^ and β_2_AR^SS^ show a marked increase in ERK phosphorylation that peaks within 2–5 min and is rapidly attenuated, suggesting a G-protein phase of activation, while β_2_AR^TYY^, shows a significantly reduced level of pERK that is prolonged and sustained (Fig. 3h, Supplementary Fig. 7a). Likewise, opto-β_2_AR and opto-β_2_AR^SS^ show a comparable temporal profile in the initial phase of ERK activation while opto-β_2_AR^LYY^ remains significantly slower and sustained (Fig. 3i, Supplementary Fig. 7b), in a similar manner to the kinetics of β_2_AR^TYY^. Further, when examined as fold increase over baseline, the effects on pERK by β_2_AR^WT^ and β_2_AR^SS^, show a marked increase in ERK phosphorylation that peaks within 2–5 min and is rapidly attenuated, while β_2_AR^TYY^ shows a reduced and sustained level of pERK (Supplementary Fig. 7c). Likewise, opto-β_2_AR^SS^ and opto-β_2_AR^LYY^ differed significantly from opto-β_2_AR, particularly in the initial stages of ERK activation (Supplementary Fig. 7d). The kinetics of both cAMP and ERK activity are remarkably similar between β_2_AR, opto-β_2_AR, and their respective G-protein-biased and arrestin-biased mutants strongly supporting the current proposed model of rapid and transient G-protein-mediated signalling, and slower sustained arrestin-mediated signalling.

### Functionally selective opto-β_2_AR mutant internalization

**Figure 4:**
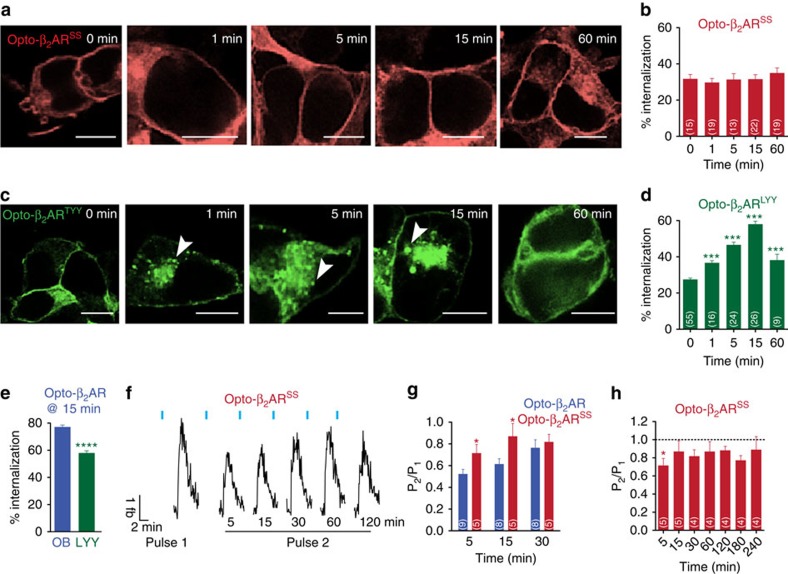
Opto-β_2_AR^SS^ and opto-β_2_AR^LYY^ internalization and desensitization. (**a**) Representative images show opto-β_2_AR^SS^–YFP (pseudocoloured red) in response to photostimulation (1 min). Scale bar, 10 μm. (**b**) Quantification of internalized opto-β_2_AR^SS^–YFP (red; (*n*=number of cells per time point). (**c**) Representative images show internalization of opto-β_2_AR^LYY^–YFP (pseudocoloured green) following light exposure (1 min). Arrowheads denote internalized receptor. Scale bar, 10 μm. (**d**) Quantification of internalized opto-β_2_AR^LYY^–YFP (green; (*n*=number of cells per time point; ***P<0.001 via One-Way ANOVA followed by Dunnett’s multiple comparison test to 0-min control). (**e**) Comparison of internalization at 15 min post photostimulation for opto-β_2_AR (dark blue; *n*=32 cells), opto-β_2_AR^LYY^ (dark green; *n*=26 cells) (****P<0.0001 via Student’s unpaired, two-tailed *t*-tests). (**f**) Representative traces show recovery from desensitization in opto-β_2_AR^SS^ expressing cells. P1 is cAMP response to initial light. P2 is cAMP response to second light pulse following different interstimulus interval. (**g**) Comparison of recovery from desensitization between opto-β_2_AR (dark blue) and opto-β_2_AR^SS^ (dark red) at low interstimulus intervals (*n*=number of of experiments; *P<0.05 via Student’s unpaired, two-tailed *t*-tests). (**h**) Quantification of opto-β_2_AR^SS^ recovery from desensitization in (**f**) (*P<0.05, paired two-tailed *t*-tests comparing P2 with P1 at each time point; (*n*=number of experiments). All data expressed as mean±s.e.m. All light pulses are 473 nm, 1 W cm^−2^.

Activity of GPCRs is usually terminated following phosphorylation via G protein-coupled receptor kinases (GRK) followed by subsequent recruitment of arrestin, leading to receptor internalization via clathrin coated pits^39^. To ascertain the characteristics of optically active, functionally selective mutant opto-β_2_ARs following desensitization we captured a time course of receptor internalization following photostimulation. Opto-β_2_AR^SS^ did not internalize at any time point tested following light stimulation, suggesting that the ability of arrestin to initiate internalization is significantly compromised in this receptor (Fig. 4a,b, Supplementary Fig. 8). Conversely, the arrestin-biased mutant, opto-β_2_AR^LYY^ was able to internalize rapidly following light stimulation, peaking at 15 min (Fig. 4c,d). In comparison to opto-β_2_AR at the 15-min time point, we see that opto-β_2_AR^LYY^ is significantly slower in reaching maximal internalization suggesting less efficient coupling with G-protein subunits that facilitate GRK recruitment (Fig. 4e). In addition, previous studies looking at β_2_AR^TYY^ also demonstrated a decrease in recruitment of arrestin which could also explain why opto-β_2_AR^LYY^ does not reach the same levels of internalization as opto-β_2_AR^11^.

In addition to demonstrating a lack of internalization for opto-β_2_AR^SS^, we determined whether opto-β_2_AR^SS^ receptors functionally desensitize. Following an initial pulse of light (P1), opto-β_2_AR^SS^ displayed a typical real-time cAMP response (Fig. 4f). Following a subsequent light pulse (P2), opto-β_2_AR^SS^ showed a mild reduction in cAMP, only at the earliest time point tested (P1 versus P2 at 5 min, *P*=0.0308 via Student’s paired *t*-test). However, overall opto-β_2_AR^SS^ did not show a significant reduction in P2-mediated cAMP (Fig. 4f–h). In comparison, opto-β_2_AR displayed a significant reduction in cAMP suggesting that the receptor functionally desensitized, and eventually recovered function within 120 min (Figs 2e–g and 4g). Opto-β_2_AR^SS^ also did not lose its ability to generate cAMP at most time points tested, unlike opto-β_2_AR, which had a dramatic loss in cAMP signalling at shorter interstimulus intervals. Due to the absence of a detectable cAMP signal at 37 °C, no functional recovery data were collected for opto-β_2_AR^LYY^. These data suggest that the putative G-protein-biased opto-β_2_AR^SS^ does not couple to arrestin as it is not internalized and desensitized following photostimulation, yet the putative arrestin-biased opto-β_2_AR^LYY^ mutant interacts with arrestin since it is robustly internalized following photostimulation.

### Photostimulation of opto-β_2_AR promotes neuronal firing

This series of *in vitro* data allowed us to illustrate that β_2_AR and opto-β_2_AR share similar properties of cAMP generation, MAP kinase activation and receptor internalization. In addition, utilizing optically active, functionally selective receptors, we gained additional insight into how these tools bias receptor function towards G-protein or arrestin-mediated signalling effects in cAMP, MAP kinase activation, and receptor internalization/desensitization. Therefore, we next examined how opto-β_2_AR functions in a biologically relevant neuronal context known to be modulated through endogenous noradrenergic activation.

**Figure 5:**
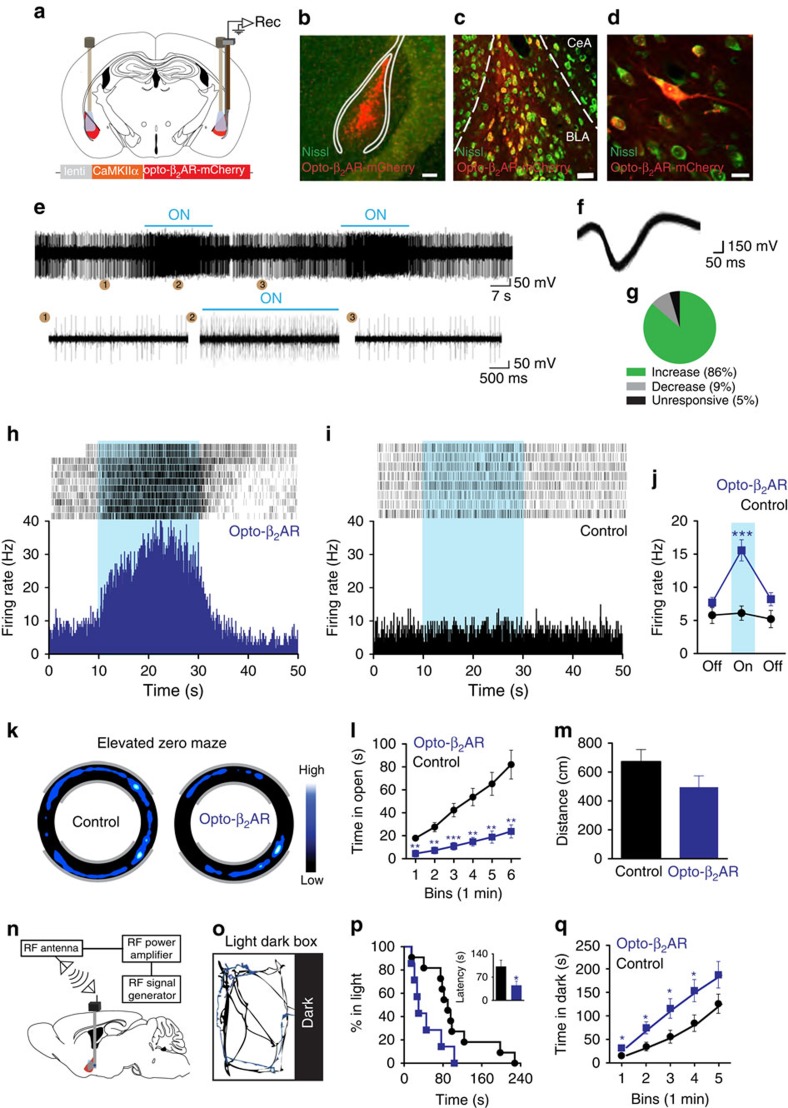
BLA neurons expressing Opto-β_2_AR promote anxiety-like behaviours. (**a**) Bilateral viral injection sites. (**b**–**d**) Lenti-CaMKIIα-opto-β_2_AR-mCherry expression in BLA (Scale bar, 50 μm (**b**), 25 μm (**c**) and 5 μm (**d**). (**e**) Representative single-unit recording (with expanded views at 1, 2 and 3) of BLA neuron reversibly increases in firing rate during light stimulation (20 s). (**f**) Representative waveforms. (**g**) Distribution of neurons that increase (green), decrease (grey) or show no change (black) in response to photostimulation. (**h**) Representative histogram shows increase in firing rate in response to photostimulation of opto-β_2_AR expressing BLA neuron. (**i**) Representative histogram shows no change in firing rate in response to photostimulation in control animals. (**j**) Opto-β_2_AR BLA expressing neurons (blue; *n*=41 units) shows reversible increase in neuronal firing rate in response to 20 s photostimulation (***P<0.001; one-way repeated measures ANOVA). Virally injected controls (black; *n*=11 units) are not light responsive. (**k**) Representative heat maps show behaviour in EZM, lighter colours indicate more time spent in a position. (**l**) Open arm cumulative time course of photostimulated opto-β_2_AR (blue, *n*=7) and control animals (black; *n*=10) (5 s off/on; **P<0.01, ***P<0.001; multiple Student’s unpaired, two-tailed *t*-tests). (**m**) Viral control (black, *n*=10) and opto-β_2_AR (blue, *n*=7) expressing animals do not show differences in total distance traveled during the EZM trial (Student’s unpaired, two-tailed *t*-test, *P*=0.1657). (**n**) Viral injection site, unilateral μ-ILED implant and brief overview of wireless transmission system. (**o**) Representative traces of control (black) and opto-β_2_AR (blue) animal in LDB. (**p**) Wireless photostimulation of mice expressing opto-β_2_AR (blue; *n*=7) in the BLA enter the dark box faster than viral controls (black; *n*=11) (*P<0.05; log-rank (Mantel-Cox) Test; inset *P<0.05 via Student’s unpaired, two-tailed *t*-test). (**q**) Opto-β_2_AR animals (blue) spend more cumulative time in the dark box than viral controls (black; *P<0.05; multiple Student’s unpaired, two-tailed *t*-tests). All data expressed as mean±s.e.m. All light pulses are 473 nm, 1 W cm^−2^.

We first determined whether photoactivation of opto-β_2_AR in cells known to express wild-type βARs, the BLA, could promote time-locked signalling effects *in vivo* and subsequent excitation of BLA neurons. We virally targeted opto-β_2_AR-mCherry to excitatory neurons under the control of the CaMKIIα promoter to the BLA (opto-β_2_AR^BLA/CaMKIIα^) (Fig. 5a–d). Utilizing a 16-channel optrode array in the BLA for single-unit extracellular recordings we delivered light stimulation (20-s pulse) *in vivo* and demonstrated a significant increase in neuronal firing of BLA neurons (Fig. 5e–j). Repeated light pulses (both 5- and 20-s constant light) also showed sustained activity over time (Supplementary Fig. 9a,b), whereas photostimulated cells in control virus (empty-vector lenti-virus) injected mice showed no effect on neuronal firing (Fig. 5i,j). In some instances, cells did not respond to light stimulation or showed inhibitory effects of photoactivation of opto-β_2_AR^BLA/CaMKIIα^ (5% and 9%, respectively; Fig. 5g, Supplementary Fig. 9c). These differences in neuronal activity may potentially be due to lack of expression of the viral construct, or lateral inhibition from local inhibitory neurons in this region^40^,^41^,^42^. In addition, we show that activation of opto-β_2_AR^BLA/CaMKIIα^ *in vivo* showed a slow onset of activation (*τ*_on_) and inactivation (*τ*_off_), similar to previous reports (Supplementary Fig. 9d)^6^. We also confirmed that the presence of opto-β_2_AR^BLA/CaMKIIα^ *in vivo* does not induce constitutive activity and alter neuronal activity as baseline firing rates between opto-β_2_AR^BLA/CaMKIIα^ (*n*=41 units) and viral control (*n*=11 units) neurons are not different (*P*=0.2593 via unpaired Student’s *t*-test) (Fig. 5j). These results demonstrate that photoactivation of opto-β_2_AR^BLA/CaMKIIα^ signalling can robustly increase neuronal activity, in a time-locked and spatially restricted manner *in vivo*.

### Photostimulation of opto-β_2_AR promotes anxiety-like behaviour

We next explored whether spatiotemporal activation of β-adrenergic signalling *in vivo* was sufficient to promote robust behavioural effects. We bilaterally expressed opto-β_2_AR^BLA/CaMKIIα^ in the BLA (Fig. 5a–d, Supplementary Figs 10a,b and 11) and photoactivated (5 s on, 5 s off) animals during two different yet widely used rodent anxiety-like behavioural models, the elevated zero maze (EZM) and the light–dark box (LDB)^43^,^44^. Photoactivation of opto-β_2_AR^BLA/CaMKIIα^ signalling in the BLA produced rapid and sustained anxiogenic-like behaviour with mice spending significantly more time in the closed arm of the EZM (Fig. 5k,l), with no changes in locomotor activity (Fig. 5m, Supplementary Fig. 10c,d). We next examined the effects of opto-β_2_AR^BLA/CaMKIIα^ activation on rapid acute anxiety-like behaviour using the LDB^45^. In this 10 min assay, rapid entry into a dark box from a light chamber and increased time spent in the dark box are measures of anxiogenesis^46^. This model consists of an enclosed dark environment with a small doorway inaccessible to fiber optic implants, we therefore utilized our recently developed wireless optogenetic approach to drive microscale inorganic light emitting diodes (μ-ILEDs)^47^,^48^ (Fig. 5n, Supplementary Fig. 12a–d). Here we developed and utilized a ‘wireless 2.0’ version, that is much smaller allowing for even more unrestricted animal activity, to remotely photoactivate opto-β_2_AR^BLA/CaMKIIα^ injected mice. In these experiments, photoactivation of opto-β_2_AR^BLA/CaMKIIα^ caused mice to rapidly enter the dark chamber as demonstrated by both a significant decrease in latency to enter the dark, and significantly more time spent in the dark during the trial (Fig. 5o–q) with no effect on locomotor behaviour (Supplementary Fig. 12e,f). Altogether, these results demonstrate that activation of opto-β_2_AR signalling *in vivo* can robustly modulate behavioural responses in βAR expressing regions. Furthermore, this can be done in a wireless manner, providing a unique method for spatiotemporal engagement of GPCR signalling *in vivo* in an unrestricted manner, such as the home cage, or other more diverse behavioural environments.

## Discussion

Manipulation of endogenous intracellular GPCR signalling *in vivo* has historically required pharmacological techniques. To some degree, optogenetics has filled this niche by allowing cell-type specificity in addition to spatiotemporal control^49^. However, a vast majority of optogenetic studies utilize light sensitive ion channels or pumps providing only binary control of neural activity. Here we show for the first time, that a chimeric rhodopsin/β_2_-adrenergic receptor (opto-β_2_AR) behaves in a kinetically similar manner, in a host of signalling readouts, to the human β_2_AR. We show that opto-β_2_AR activates GPCR signalling in a power dependent manner, mimicking the concentration dependence of isoproterenol on β_2_AR. Importantly, we also report that the kinetic responses of these two receptors are similar and that opto-β_2_AR not only internalizes following stimulation, but also functionally desensitizes, sharing the same kinetics as β_2_AR. Taken together these data strongly support using opto-β_2_AR as a tool to mimic β_2_AR activity (see summary data in Supplementary Table 1). In addition to modelling β_2_AR at the receptor level, incorporation of opto-β_2_AR as a tool allows for modelling the kinetics of endogenous NE release as the activation and deactivation of this receptor is controlled instantly through optogenetic techniques. In contrast, small molecules may confound analysis at the circuit level, as drug clearance becomes an issue and systemic half-life makes conclusions regarding kinetics of behavioural onset/offset difficult to interpret. Conversely, it must be taken into account that opto-β_2_AR is not identical to β_2_AR, and it is not currently known whether the trafficking and recycling pathways are the same *in vivo*. Future studies will need to further characterize the intracellular dynamics of opto-β_2_AR *in vivo* using real-time imaging approaches.

In addition to showing the utility of optically activated GPCRs, we show here for the first time, optically active, functionally selective GPCRs. The concept of functional selectivity or biased agonism is becoming increasingly important to understand GPCR biology and in the development of novel therapeutics^9^,^50^,^51^. Here we show two functionally distinct receptors: the arrestin-biased, opto-β_2_AR^LYY^, and the G-protein-biased, opto-β_2_AR^SS^. Through an array of biochemical analyses we show that opto-β_2_AR^LYY^: mobilizes less cAMP, shows reduced and prolonged activation of ERK and internalizes in response to light stimulation in comparison to opto-β_2_AR. Conversely, opto-β_2_AR^SS^: shows enhanced and transient cAMP signalling, shows elevated transient activation of ERK, lacks internalization, and shows little desensitization as compared with opto-β_2_AR. These optically sensitive, functionally selective GPCRs provide the advantages of not requiring a biased ligand and have direct spatiotemporal control over receptor activation and deactivation (see summary data in Supplementary Table 2).

The information gathered from *in vitro* studies regarding the intracellular characteristics of opto-β_2_AR in a heterologous expression system provided confidence that opto-β_2_AR closely mimics the pharmacological properties of β_2_AR. To expand on this potential utility, we further validated this approach *in vivo*, in a biologically relevant anxiety circuit known to be modulated through noradrenergic activation, the BLA. Given that the BLA is composed mostly of excitatory neurons^52^,^53^, we packaged opto-β_2_AR under the control of the CaMKIIα promoter to drive robust expression in the BLA (opto-β_2_AR^BLA/CaMKIIα^). Photostimulation of opto-β_2_AR^BLA/CaMKIIα^ *in vivo* altered the baseline firing properties of BLA neurons and revealed a heterogeneous population of cells. While the majority of cells increased firing rate, some exhibited no change and some showed a reduction in firing rate. Utilizing traditional pharmacological approaches would not allow for the isolation of a cell type within a given anatomical region and hence the roles of individual cell types, and adrenergic receptor subtypes were not previously possible.

When opto-β_2_AR^BLA/CaMKIIα^ was activated *in vivo*, mice exhibited an anxiety-like phenotype. Anxiogenesis was demonstrated in two commonly used models of anxiety-like behaviour in rodents, the EZM^43^ and the LDB^46^. Utilization of the LDB was only possible due to new wireless optogenetic technology recently developed^47^,^48^. Here we also demonstrate for the first time, real-time wireless control of GPCR signalling. Opto-β_2_AR^BLA/CaMKIIα^ expressing mice, when stimulated wirelessly, displayed an anxiety-like phenotype in the LDB assay. This technology allowed us to use a common model of anxiogenic behaviour, but also sets the stage for important future work utilizing wireless manipulation of GPCR signalling *in vivo* allowing for a large expansion of GPCR-mediated behaviours that can be paired with current optogenetic techniques. That being said, optogenetics comes with certain caveats. It is possible that photostimulation in the BLA also activates axon collaterals, and hence confound subsequent behavioural output, or that more complex cell types and expression patterns are needed to truly hone in on β_2_AR signalling in real-time, *in vivo*. However, this is a unique first approach, and as additional mouse genetic tools and targeting schemes become available^54^, further isolation of cell types and GPCR signalling in neural circuits will become increasingly possible.

Taken together our data demonstrate that chimeric, optically active GPCRs can behave in a similar manner to their endogenous counterparts, making them particularly useful for both *in vitro* and *in vivo* applications. Future studies will utilize these tools to engage the diversity of GPCR signalling *in vivo* and determine if spatiotemporal control of biased signalling promotes a series of pluridimensional behavioural phenotypes. In this report, we were able to show that the integration of the excitatory noradrenergic influence in the BLA is mediated via activation of β-adrenergic pathways that ultimately promotes anxiogenic-like behavioural states. Some exciting additional extensions include using them for examining the role of signalling inside cells, since the light used to activate these receptors can penetrate the cell membrane. Recent work from von Zastrow and colleagues has shown that Gαs-coupled receptors have multi-phasic signalling properties, one that is membrane bound, and another that occurs from endosomes^55^,^56^. Other uses of these receptors include a means to better understand the process of G-protein activation without confounds of ligand binding. These findings have broad implications for our understanding of the mechanisms of GPCR signalling *in vivo* and in the development of novel therapeutics that depend on interactions with GPCRs.

## Methods

### Chemicals

Isoproterenol (10 mM dimethyl sulfoxide (DMSO) stock), forskolin (10 mM DMSO stock) and NF 449 (10 mM water stock) were obtained from Tocris Biosciences. 9-*cis*-retinal (10 mM DMSO stock and shielded from light) was obtained from Sigma. Vehicle controls were used in all cases.

### Light stimulation

All light stimulation was constant at 473 nm, at powers and pulse lengths indicated in figure legends. Light was delivered via a 100-mW, 473-nm diode-pumped solid-state laser (OEM Laser Systems)^4^,^57^.

### Cell culture

HEK293 cells (ATCC, CRL-1573) were grown in DMEM supplemented with 10% fetal bovine serum containing 1 × pen/strep (Invitrogen) and maintained at 37 °C in a humidified incubator with 5% CO_2_. Stable HEK293 cell lines expressing pcDNA3.1 containing opto-β_2_AR, β_2_AR and their respective mutants were generated by transfecting HEK293 cells with identical amounts of cDNA (10 μg in 100-mm dishes) for 4 h using JetPrime (Polyplus) reagent per manufacturer’s instructions. Cells were placed under selective pressure with G418 (400 μg ml^−1^) and FACS (Washington University FACS Sorting Facility) sorted for equal yellow fluorescent protein (YFP) fluorescence to ensure equivalent receptor expression. All experiments utilizing opto-β_2_AR and its mutants were performed in the dark in the presence of 1 μM 9-*cis*retinal (Sigma).

### Site-directed mutagenesis

Human adrenergic receptor beta 2 (ADRB2), Gene Bank Accession Number: NM_000024.3 was purchased from cDNA.org.

β_2_AR–YFP fusion protein was created using human ADBR2 as a template in a high fidelity PCR using the following primers: with primers EcoRI-β_2_AR-forward (5′-AGT GTG GTG GAA TTC GAT TAT CCA CC-3′) and XhoI-β_2_AR-reverse (5′-CCT CTA GAC TCG AGT aAC AGC AGT GA-3′) with the stop codon mutated to leucine. The PCR product was then digested and cloned into the 5′ EcoRI and 3′ XhoI sites of pcDNA3–YFP (Addgene Plasmid 13033).

β_2_AR^SS^–the ‘G-protein biased’ mutation (S355A/S356G) was created using human ADBR2 as a template in a high fidelity PCR using the following primers: internal primers containing point mutations in lower case, forward (5′-TAT GGG AAT GGC TAC gCC gcC AAC GGC AAC ACA GG-3′) and reverse (5′-CCT GTG TTG CCG TTG gcG GcG TAG CCA TTC CCA TA-3′) with external primers EcoRI-β_2_AR-forward (5′-AGT GTG GTG GAA TTC GAT TAT CCA CC-3′) and XhoI-β_2_AR-reverse (5′-CCT CTA GAC TCG AGT aAC AGC AGT GA-3′) with the stop codon mutated to leucine. The PCR product was then digested and cloned into the 5′ EcoRI and 3′ XhoI sites of pcDNA3–YFP (Addgene Plasmid 13033).

β_2_AR^TYY^–the ‘arrestin biased’ mutation was obtained from Robert Lefkowitz (Duke University). We created a fusion protein between β_2_AR^TYY^ and YFP. β_2_AR^TYY^ was amplified via high fidelity Taq with the following primers: EcoR1-β_2_AR^TYY^-forward (5′-TAC AAG GAC GAT GAa ttC atg GGG CAA CCC GGG AAC GGC A-3′) and XhoI-β_2_AR^TYY^-reverse (5′-GCG GCC GTT ctc gag tgc CAG CAG TGA GTC ATT TGT ACT-3′) with stop codon changed to an alanine. The PCR product was then digested and cloned into the 5′ EcoRI and 3′ XhoI sites of pcDNA3–YFP (Addgene Plasmid 13033).

Opto-β_2_AR^SS^–the ‘G-protein biased’ mutation (S362A/S363G) was created using a COBALT alignment against human β_2_AR (S355A/S356G). Opto-β_2_AR was obtained from Karl Deisseroth (Stanford University) and used as the template in a high-fidelity PCR using the following primers: internal primers containing point mutations in lower case, forward (5′-TCC AAA GCG TAC GGA AAT GGC TAT gCA gga AAC AGC AAC GGA AAG ACT GAT TAT-3′) and reverse (5′-ATA ATC AGT CTT TCC GTT GCT GTT tcc TGc ATA GCC ATT TCC GTA CGC TTT GGA-3′) with external primers HindIII- opto-β_2_AR^WT^-forward (5′-CCA AGC TGG CTA GTT AAG CTT GCC ACC-3′) and NotI–opto-β_2_AR^WT^–rev (5′-GCT CAC GGC GGC CGC GGC CGG AGC GAC-3′). PCR product was then digested and cloned into the 5′ HindIII and 3′ NotI sites of pcDNA3.1–YFP (generously provided by Deisseroth Lab).

Opto-β_2_AR^TYY^, the ‘arrestin biased’ mutation was created using a COBALT alignment between opto-β_2_AR and β_2_AR^TYY^ point mutations: L72F, Y136G, Y224A were generated using the opto-β_2_AR^WT^ as a template in a high-fidelity PCR using the following primers: opto-β_2_AR^L72F^-forward (5′-CTC CAA ACC GTG TTt AAC TAC ATA CTC CTT-3′), opto-β_2_AR^L72F^-reverse (5′-AAG GAG TAT GTA GTT aAA CAC GGT TTG GAG-3′); opto-β_2_AR^Y136G^-forward (5′-TTG GCC ATA GAG AGG ggC GTG GTG GTC ACA-3′), opto-β_2_AR^Y136G^-reverse (5′-TGT GAC CAC CAC Gcc CCT CTC TAT GGC CAA-3′); opto-β_2_AR^Y224A^-forward (5′-ATC TTT TTC TGT gcC GGC AGG GTG TTC CAG-3′), opto-β_2_AR^Y224A^-reverse (5′-CTG GAA CAC CCT GCC Ggc ACA GAA AAA GAT-3′) with the external primers HindIII- opto-β_2_AR^WT^-forward (5′-CCA AGC TGG CTA GTT AAG CTT GCC ACC-3′) and NotI- opto-β_2_AR^WT^-rev (5′-GCT CAC GGC GGC CGC GGC CGG AGC GAC-3′). PCR product was then digested and cloned into the 5′ HindIII and 3′ NotI sites of pcDNA3.1–YFP (generously provided by Deisseroth Lab).

All mutations were confirmed by DNA sequencing (AGCT Inc., Wheeling, IL).

### Real time cAMP assay

Stable HEK cell lines containing opto-β_2_AR, β_2_AR and their respective mutants were transfected with the pGloSensor-22F cAMP plasmid (Promega E2301) using JetPrime (Polyplus) transfection reagent per manufacturer’s instructions. Stable co-transfected cells were maintained under both G418 (400 μg ml^−1^) and hygromycin (200 μg ml^−1^) selective pressure. The day before an experiment, cells were plated on 96-well tissue culture treated plates (Costar) and allowed to recover overnight at 37 °C, 5% CO_2_. Optimal results were obtained when β_2_AR (and respective mutants) were plated at 20 K cells per well and when opto-β_2_AR (and respective mutants) were plated at 100 K cells/well. The next day, media was replaced with 2% GloSensor reagent (Promega) suspended in CO_2_-independent growth medium (Gibco) and incubated for 2 h at 37 °C or 25 °C depending on experiment. For real time cAMP, a baseline was first obtained with no treatment by recording relative luminescent units (RLUs) every 6 s for 1 min using a SynergyMx microplate reader (BioTek; Winooski VT; USA). Drug or light would then be used to stimulate the cells, and subsequent RLUs recorded every 6 s for 5–10 min depending on experiment. For data expressed as cAMP (fold baseline), RLUs for 1 min of baseline were averaged and all subsequent RLUs were then divided by this average. For data expressed as cAMP (% max), raw RLUs were entered into GraphPad Prism (v5.0d, GraphPad Software, San Diego California USA) and the normalization function used to assign the lowest RLU a value of 0% and the highest RLU a value of 100%. Time constants were calculated in GraphPad Prism using one-phase association (*Y*=Y0+(Plateau−Y0) × (1−exp(−K × *x*))) and one-phase decay (Y=(Y0−Plateau) × exp(−K × *X*)+Plateau) nonlinear regression analyses yielding a time constant value (*τ*).

### Concentration/power response curves

For β_2_AR experiments, baseline relative luminescence recordings were taken and cells exposed to varying concentrations of isoproterenol in serial half log dilutions diluted from 10 mM stock in DMSO. Raw RLUs were normalized to the peak response evoked by isoproterenol and represented as cAMP (% max). Subsequent concentration response curves were fit using standard nonlinear regression to obtain EC_50_ values using GraphPad Prism and expressed as mean±s.e.m., with triplicate data points averaged per experiment with a total of six individual experimental replicates. For opto-β_2_AR experiments, individual wells were exposed to a 5-s blue light pulse (473 nm) at varying powers to generate a power response curve with data normalized to maximal cAMP response. Subsequent power response curves were fit with standard nonlinear regression to obtain EP_50_ values using GraphPad Prism. Data are expressed as mean±s.e.m.

### Recovery from desensitization

Opto-β_2_AR^WT^ and opto-β_2_AR^SS^ cells grown in 96-well plates were individually exposed to a single 5-s blue light pulse (473 nm, 1 W cm^−2^) called P1 (pulse 1) with the subsequent cAMP response recorded. After varying interstimulus intervals (0, 2.5, 5, 15, 30, 60, 120, 180 and 240 min) each well was then re-exposed to a second light pulse (P2), and subsequent cAMP response recorded. Peak RLU for P2 were then divided by peak RLU for P1. These points were then fit with a one-phase association (Y=Y0+(Plateau−Y0) × (1−exp(−K × *x*))) curve in GraphPad Prism to obtain a time constant of recovery from desensitization (*τ*_rec_). Data are expressed as mean±s.e.m.

### Immunoblots

Western blots for phospho-MAPKs were performed as described previously^58^. Cells were grown overnight in 6- or 12-well plates, then serum-starved a minimum of 4 h before treatment to avoid serum growth factor-induced MAPK activation. Cells were treated at various time points at 37 °C and then collected in lysis buffer (50 mM Tris-HCl, 300 mM NaCl, 1 mM EDTA, 1 mM Na3VO4, 1 mM NaF, 10% glycerol, 1% Nonidet P-40, 1:100 of phosphatase inhibitor mixture set 1 (Calbiochem), and 1:100 of protease inhibitor mixture set 1 (Calbiochem) on ice. Lysates were sonicated for 15 s, centrifuged for 20 min (14,000 rcf at 4 °C), then stored at −20 °C. Protein concentration was determined by Pierce BCA (Thermo Scientific) with bovine serum albumin as the standard. Each gel contained the same amount of total protein and varied between 20–40 μg, depending on experiment. Nondenaturing 10% bisacrylamide precast gels (Invitrogen) were run at 180 V for 1 h. For determination of molecular weights, pre-stained molecular weight ladders (Life Technologies; Novex Sharp Protein Standard; LC5800) were loaded along with protein samples. Blots were transferred to nitrocellulose (Whatman, Middlesex, UK) for 1.5 h at 30 mV, blocked in 5% bovine serum albumin in tris-buffered saline (TBS) for 1 h, incubated overnight at 4 °C with goat anti-rabbit phospho-ERK 1/2 (Thr-202/Tyr- 204) antibody (1:1,000, Cell Signaling) and mouse β-actin (1:20,000, Abcam). Membranes were then washed 4x for 10 min in TBST (Tris-buffered saline, 1% Tween 20) and then incubated with the IRDyeTM 800 (1:5,000, donkey anti-rabbit) and 700 (1:20,000, donkey anti mouse) conjugated affinity purified IgG in a 1:1 mixture of 5% milk/TBS and Li-Cor blocking buffer (Li-Cor Biosciences, Lincoln, NE) for 1 h at room temperature in the dark. Membranes were then washed three times for 10 min in TBST then once for 10 min in TBS to remove Tween. Immunoblots were scanned using the Odyssey infrared imaging system (Li-Cor Biosciences). Band intensity was measured using Odyssey software following background subtraction and integrated intensity measured for each band in high-resolution pixels. All pERK bands were normalized to β-actin, as an equal protein loading control. For data expressed as pERK (fold baseline), raw pERK/actin values were normalized to the 0-min time point. For data expressed as pERK (% max), raw pERK/actin values were entered into GraphPad Prism (v5.0d, GraphPad Software, San Diego CA, USA) and the normalization function used to assign the lowest pERK/actin a value of 0% and the highest raw pERK/actin a value of 100%. Data are expressed as mean±s.e.m. Concentration-response data were fit using nonlinear regression in GraphPad Prism. Positive controls are cell lysates obtained from HEK293 cells stably expressing the Nociceptin/Orphanin FQ Opioid Peptide Receptor (NOPR) harvested following a 5-min incubation in nociceptin (1 μM)^58^. These independent positive controls are to ensure successful execution of western blots and in no way affect the data presented. Full unaltered scans of all western blot images with corresponding molecular markers can be found in Supplementary Figs 13–16.

### On cell western

On cell westerns were performed following previously published protocols^37^,^38^. HEK293 cells stably expressing opto-β_2_AR, opto-β_2_AR^SS^, opto-β_2_AR^LYY^ and untransfected control HEK293 cells were plated on 24-well tissue culture treated plates at 200 K cells/well and grown in DMEM containing 10% fetal bovine serum and penicillin/streptomycin at 37 °C in 5% CO_2_. Plate was placed on ice, media removed and cells immediately fixed with 4% paraformaldehyde for 30 min at room temperature. Cells were washed five times for 30 min in PBS, blocked for 90 min in Li-COR Odyssey Blocking Buffer at room temp with gentle rocking. Cells were incubated overnight at 4 °C in mouse rhodopsin antibody (4D2) (Novus NBP1–48334) diluted 1:1,000 in Odyssey Blocking Buffer. Wash five times in Tris-buffered saline containing 0.1% Tween-20 (TBST) for 30 min. Incubate in Li-Cor IRDye 680RD (donkey α mouse), diluted 1:1,000 in Odyssey buffer+0.1% TWEEN-20 at room temperature for 1 h. Wash five times in TBST for 30 min. After final wash, remove solution from wells, tap or blot gently on paper towels to remove traces of wash buffer and scanned using the Odyssey infrared imaging system (Li-Cor Biosciences). Well intensity was measured using Odyssey software following background subtraction and integrated intensity measured for each well in high-resolution pixels. Data were analysed in GraphPad Prism and are expressed as mean±s.e.m.

### Receptor internalization

β_2_AR–YFP, opto-β_2_AR^WT^–YFP, opto-β_2_AR^SS^–YFP and opto-β_2_AR^LYY^–YFP were plated on collagen/poly-D-lysine coverslips in 24-well plates at 50 K cells per well and placed in 37 °C, 5% CO_2_ humidified incubator overnight. Following treatment the following day, cells were washed three times with PBS and then fixed in 4% paraformaldehyde for 20 min, washed three times in PBS, washed twice in PB and then mounted with VECTASHIELD (Vector Laboratories, Burlington, CA). All imaging was performed within the Washington University Pain Center Confocal Imaging Center. Images, cells, and treatment groups were chosen and analysed in a blinded fashion. Semi-quantitative analysis of internalization was calculated as previously described using Metamorph (Molecular Devices, CA, USA) analysis algorithm for pixel intensity measurements of internalized fluorescence measures^58^. To determine internalized percentages, equal cell shapes and sizes were always chosen; concentric circles around the fluorescence, background internal fluorescence (untreated controls) or internalized (treated) portions of the entire cell were drawn in Metamorph, integrated pixel intensities were recorded for each using the Metamorph algorithm for integrating intensity and internalized receptors were calculated using: Inside F/Total F to produce the internalization ratio. Data are expressed as mean±s.e.m.

### *In vitro* calcium imaging

Cells were plated on collagen/poly-D-lysine glass coverslips, loaded with Fura-2 acetoxymethyl ester (2.5–5 mM), and incubated for 60 min at room temperature in 1.5 mM of pluronic acid (Molecular Probes, Eugene, OR) in a HEPES-buffered saline (2 mM Ca^2+^). Coverslips were placed in a laminar flow perfusion chamber (Warner Instrument Corp.) and constantly perfused with HEPES-buffered saline (2 mM Ca^2+^). Images of Fura-2-loaded cells with the excitation wavelength alternating between 340 and 380 nm were captured. Following subtraction of background fluorescence, the ratio of fluorescence intensity at the two wavelengths was calculated. Ratio levels were analyzed using MetaFluor (Universal Imaging Corporation).

### Animals

Adult (25–35 g or 2 to 3 months old) male C57BL/6J mice were used in all *in vivo* experiments. Mice were group-housed, given access to food and water *ad libitum* and maintained on a 12 h:12 h light:dark cycle. All animals were held in a facility in the lab 1 week before surgery, post-surgery and throughout the duration of the behavioural assays to minimize stress from transportation and disruption from foot traffic. All procedures were approved by the Animal Care and Use Committee of Washington University and conformed to US National Institutes of Health guidelines.

### Viral preparation

Plasmid encoding pLenti-CaMKIIα-opto-β_2_AR-mCherry (final titer 4.8 × 10^8^ IU ml^−1^) was obtained from Deisseroth Laboratory at Stanford University and packaged at the WUSTL Hope Center Viral Vector Core. Lenti-PGK-GFP (viral control; final titer 1.3 × 10^8^ IU ml^−1^) was provided by the WUSTL viral core facility. AAV5-CaMKIIα-HA-GSD-IRES-mCitrine (final titer 3 × 10^12^ virus molecules per ml) and AAV5-CaMKIIα-eGFP (final titer 5 × 10^12^ virus molecules per ml) were obtained from University of North Carolina Gene Therapy Center Vector Core and Virus Vector Core.

### Stereotaxic surgery

Mice were anaesthetized in an induction chamber (5% isoflurane) and placed in a stereotaxic frame (Kopf Instruments, Model 1900) where they were maintained at 1–2% isoflurane throughout the procedure. Following craniotomy mice were injected bilaterally with 1.2 μl of either lenti-EF1α-GFP or lenti-CaMKIIα-optoβ_2_AR-mCherry in the BLA at stereotaxic coordinates: −1.3 mm posterior to bregma; ±2.9 mm lateral to bregma and −4.9 mm ventral to bregma. For wireless μ-ILED BLA studies, animals were injected unilaterally, not bilaterally. Mice were then implanted with chronic fiber optic implants or μ-ILED wireless devices with coordinates adjusted from viral injection to: −1.3 mm posterior to bregma; ±2.9 mm lateral to bregma and −3.9 mm ventral to bregma. For bio-dissolvable samples, the device was implanted at the desired target, ACSF was applied to the portion of the device that remained outside of the skull to facilitate dissolution of the adhesive, and then the epoxy needle was removed after a delay of 15 min^47^,^48^. The fiber optic implants and wireless μ-ILED devices were secured using two bone screws (CMA, 743102) and affixed with TitanBond (Horizon Dental Products) and dental cement (Lang Dental)^48^. Mice were allowed to recover for at least 3–6 weeks before behavioural testing; this interval also permitted optimal viral expression.

### *In vivo* electrophysiology

Spontaneous single unit activity was recorded following previous published protocols^47^,^57^. Briefly, mice were lightly anesthetized (1% isoflurane), placed in a stereotactic frame and two skull screws were placed on either side of the midline to ground the electrode array. The recording apparatus consisted of a 16-channel (35-μm tungsten wires, 150-μm spacing between wires, 150-μm spacing between rows, Innovative Physiology) electrode array. This array was epoxied to a fiber optic and lowered into the BLA (stereotaxic coordinates from bregma: −1.3 mm (AP),±2.9 mm (ML) and −4.9 mm (DV). Spontaneous and photostimulated neuronal activity was recorded from each electrode, bandpass-filtered with activity between 250 and 8,000 Hz, and analysed as spikes. Voltage signals were amplified and digitally converted using Omniplex and PlexControl (Plexon). For opto-β_2_AR^WT^, 5 s constant light, followed by 5-s no light was repeated for 12 cycles or 20-s constant light (on) followed by 1-min recovery with no light (off) was repeated for 12 cycles. Principle component analysis and/or evaluation of *t*-distribution with expectation maximization were used to sort spikes using Offline Sorter (Plexon). Cells were considered excited if there was than a 10% increase in baseline firing frequency, and inhibited if there was > 10% decrease in baseline firing frequency in the presence of constant photostimulation.

### Wireless powering and RF scavenger for wireless optogenetics

Wireless powering of the μ-ILED devices was performed following previously published protocols^47^,^48^. The wireless power transmitter includes an RF signal generator (Agilent N5181A), a power supply (Agilent U8031A), a RF power amplifier (Empower RF Systems 1119-BBM3K5KHM), an RF signal splitter (RF Lambda RFLT2W0727GN), and two panel antennas (ARC Wireless ARC-PA2419B01). The RF signal generator is internally modulated to delivery sufficient power to light the μ-iLEDs at the given stimulation protocol (10 Hz, 50-ms pulse widths). The RF power amplifier that is powered by the power supply enlarges the modulated RF signal from the RF signal generator. The RF power is then transmitted from the panel antenna to the headstage power harvesters. The RF signal generator has a power output from −10 to 0 dBm at 1.5 GHz, optimized daily to ensure equivalent light power throughout the space of the LDB assay. Mice with chronically implanted μ-ILED devices were acutely connected to the headstage power harvesters immediately before any wireless photostimulation.

### Behaviour

Behavioural assays were performed in a special sound attenuated room maintained at 23 °C. Lighting was measured and stabilized at ∼4 lux for anxiety tests and ∼200 lux for place testing. All behavioural apparatuses were cleaned with 70% ethanol in between animals. In each assay, animals received constant photostimulation throughout the entire trial. For all behavioural experiments, lenti-EF1α-GFP and lenti-CaMKIIα-optoβ_2_AR-mCherry received 5 s of constant photostimulation (473 nm) followed by 5 s of no light throughout the entire trial. Movements were video recorded and analyzed using Ethovision Software.

*Elevated zero maze*. The EZM (Harvard Apparatus) was made of grey plastic, 200 cm in circumference, comprised of four 50-cm sections (two opened and two closed). The maze was elevated 50 cm above the floor and had a path width of 4 cm with a 0.5 cm lip on each open section. Animals were connected to cables coupled to a function generator, positioned head first into a closed arm, and allowed to roam freely for 6 min. Mean open arm time was the primary measure of anxiety-like behaviour.

*Light/dark box*. The LDB was a 50 × 50 cm square plexiglass enclosure with a 16.5 cm × 49 cm dark insert. For testing, animals were connected to wireless harvester and placed into the corner of the open enclosure and allowed to roam freely for 10 min.

### Immunohistochemistry

At the conclusion of behavioural testing, mice were anaesthetized with sodium pentobarbital and transcardially perfused with ice cold PBS, followed by 4% phosphate-buffered paraformaldehyde following previously published protocols^47^. Brains were removed, post-fixed overnight in paraformaldehyde, and saturated in 30% phosphate-buffered sucrose. Sections of 30 μm were cut, washed in 0.3% Triton X100/5% normal goat serum in 0.1 M PBS, stained with fluorescent Nissl stain (1:400 Neurotrace, Invitrogen, Carlsbad, CA) for 1 h, and mounted onto glass slides with Vectashield (Vector Laboratories, Burlingame, CA). opto-β_2_AR expression was verified using fluorescence (Olympus, Center Valley, PA) and confocal microscopy (Leica Microsystems, Bannockburn, IL). Images were produced with Leica Application Suite Advanced Fluorescence software. Animals that did not show targeted expression were excluded from analyses.

### Statistics/data analysis

All data are expressed as mean±s.e.m. Data were normally distributed, and differences between two groups were determined using independent Students’ two-tailed, unpaired or paired *t*-tests as appropriate. Differences between multiple groups were determined via one-way or two-way analysis of variances (ANOVAs) followed by *post hoc* Bonferroni or Dunnett’s multiple comparisons if the main effect was significant at *P*<0.05. Statistical significance was taken as *P<0.05, **P<0.01, ***P<0.001, ****P<0.0001 and all analyses were conducted using Prism 5.0 (GraphPad). Grubbs’ test was used to remove any statistical outliers.

## Additional information

**How to cite this article:** Siuda, E. R. *et al*. Optodynamic simulation of β-adrenergic receptor signalling. *Nat. Commun.* 6:8480 doi: 10.1038/ncomms9480 (2015).

## Supplementary Material

Supplementary Information Supplementary Figures 1-16, Supplementary Tables 1-2 and Supplementary References
