# Supplementary material for: Optodynamic simulation of β-adrenergic receptor signalling
Source: Nat Commun. 2015 Sep 28;6:8480. doi: 10.1038/ncomms9480 (PMC4588095; doi:10.1038/ncomms9480)
Supplement: Supplementary Information — Supplementary Figures 1-16, Supplementary Tables 1-2 and Supplementary References [file ncomms9480-s1.pdf]

Supplementary Figure 1

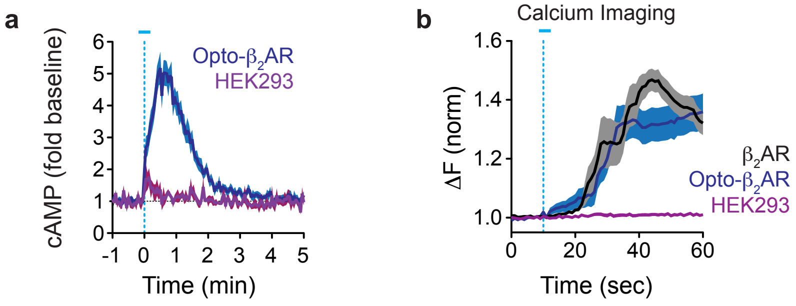

Supplementary Figure 1: Opto-β_2AR and β_2AR share similar cAMP and pERK signaling mechanisms. (a) cAMP is activated in opto-β_2AR (blue, n = 14 experiments) in response to light (5 sec, dotted line). Purple trace show HEK-pGlo cells in response to the same light stimulus (n = 4 experiments) (mean = solid line, SEM = shaded area). **(b)** Intracellular Ca^{+2} response of opto-β_2AR (blue; 1 min light; n = 13 cells), β_2AR (black; 1 μM isoproterenol; n = 7 cells) and HEK293 cells (purple; 1 min light; n = 9 cells) co-transfected with CNGA (mean = solid line, SEM = shaded area). All data are expressed as mean ± SEM. All light pulses are 473 nm at 1 W/cm^2 unless otherwise noted.

Supplementary Figure 2

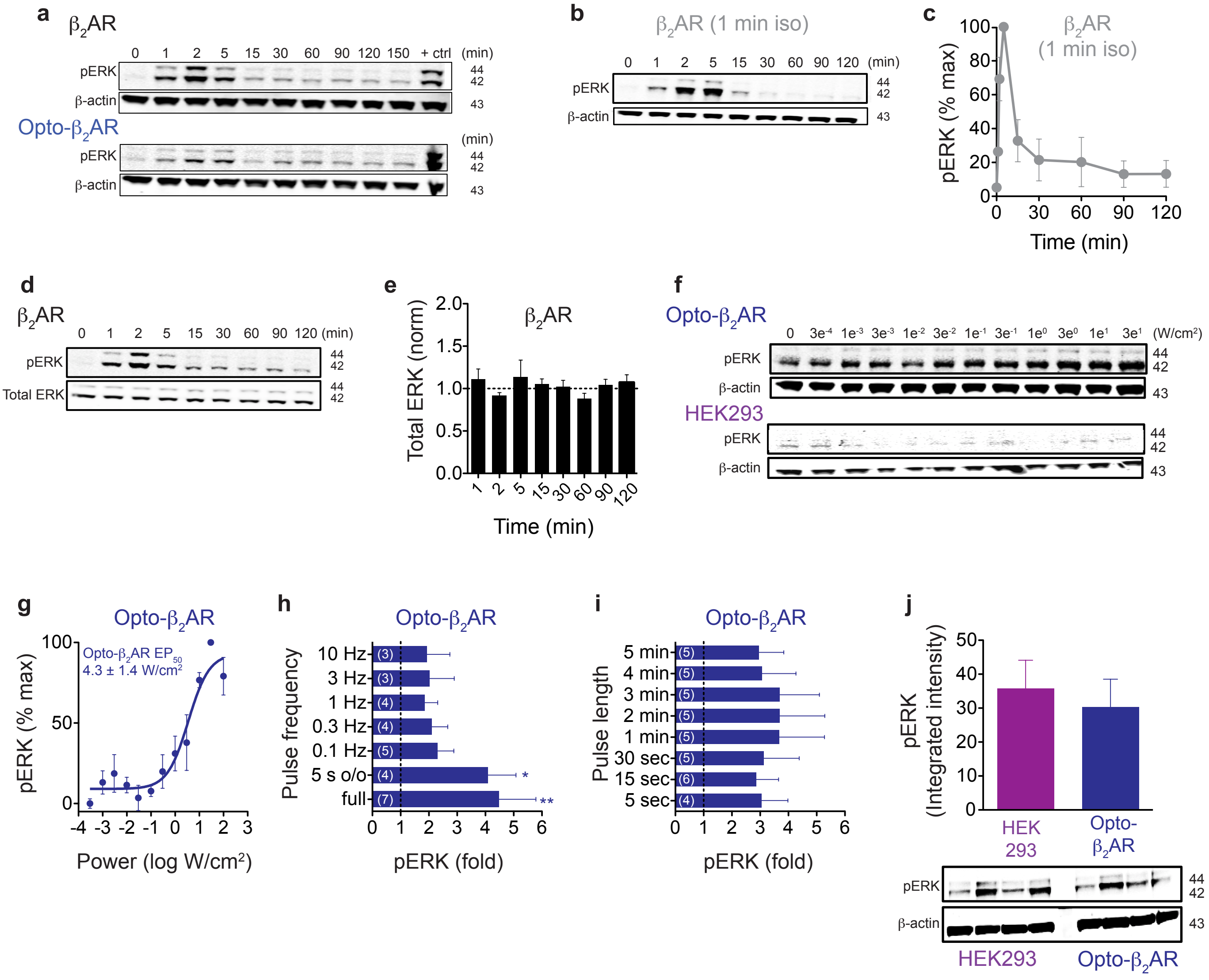

Supplementary Figure 2: Opto-β$_2$AR and β$_2$AR share similar cAMP and pERK signaling mechanisms. (a) Full pERK time course blots from **Figure 1f,g. (b)** pERK time course following 1 min isoproterenol (1 μM) wash in β$_2$AR (n = 2 experiments). **(d)** pERK time course in β$_2$AR compared to total ERK. **(e)** Quantification of total ERK integrated intensity normalized to 0 min time point (n = 4 experiments). **(f)** Representative opto-β$_2$AR and HEK293 control pERK immunoblots in response to increasing light power. **(g)** pERK power response curve of opto-β$_2$AR in response to increasing light power (EP_{50} = 4.3 ± 1.4 W/cm^2; n = 4 experiments). **(h)** Continuous light (full) or 5 sec light pulses on/off [o/o] for 1 min show significantly elevated levels of pERK from baseline in opto-β$_2$AR (*$p < 0.05$, **$p < 0.01$ via One-Way ANOVA followed by Dunnett's multiple comparison test to no treatment control; n = (#) experiments). **(i)** Different lengths of continuous light show similar increases in pERK in opto-β$_2$AR (blue) (n = (#) experiments). **(j)** Raw pERK integrated intensity values from same gel of unstimulated HEK293 cells with opto-β$_2$AR (blue) and without (purple) (n = 4 experimental replicates). All data are expressed as mean ± SEM. All light pulses are 473 nm at 1 W/cm^2 unless otherwise noted.

Supplementary Figure 3

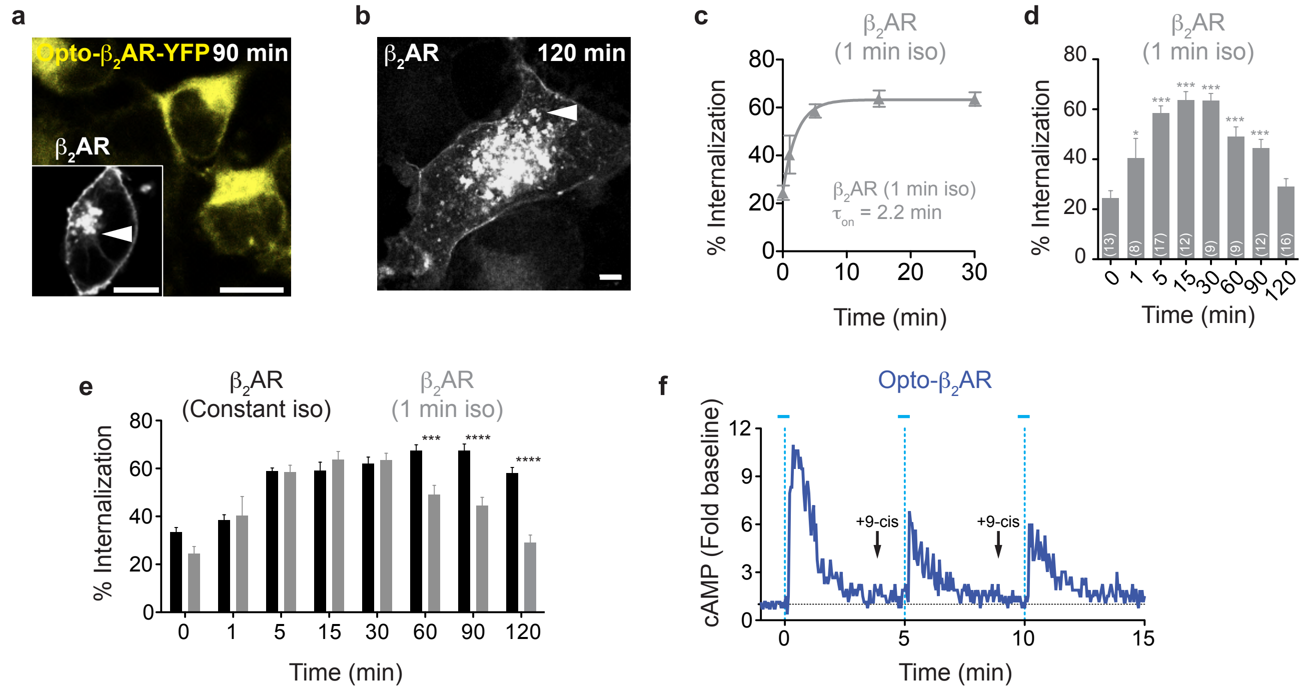

Supplementary Figure 3: Opto-β$_2$ARs functionally internalize and recover from desensitization. (a) Representative image shows internalization of opto-β$_2$AR-YFP receptors in response to light stimulation (1 min pulse) fixed at 90 min time point post stimulation. Notice lack of internalized receptor (scale bar = 10 µm). Inset shows internalization of β$_2$AR-YFP (colorized to black and white) in response to 1 µM isoproterenol at same time point. Arrowhead shows punctate internalized receptor (scale bar = 10 µm). **(b)** Representative image shows internalization of β$_2$AR-YFP (colorized to black and white) receptors in response to isoproterenol (1 µM) fixed 120 min time point post stimulation (scale bar = 5 µm). **(c)** Quantification of internalization in β$_2$AR-YFP (grey; τ_{on} = 2.2 min). **(d)** Percent internalization for β$_2$AR-YFP (*p < 0.05, ***p < 0.001 via One-Way ANOVA followed by Dunnett's multiple comparison test to 0 min control; (n = (#) cells per time point). **(e)** Comparison of Figure 2d (constant iso; black) and Supplementary Figure 2d (1 min iso; grey) (*** p < 0.001, **** p < 0.0001 via Two-Way ANOVA followed by Bonferroni's multiple comparison test). **(f)** Addition of 9-cis-retinal (1 µM) to wells before each light pulse. All light pulses are 473 nm at 1W/cm^2.

Supplementary Figure 4

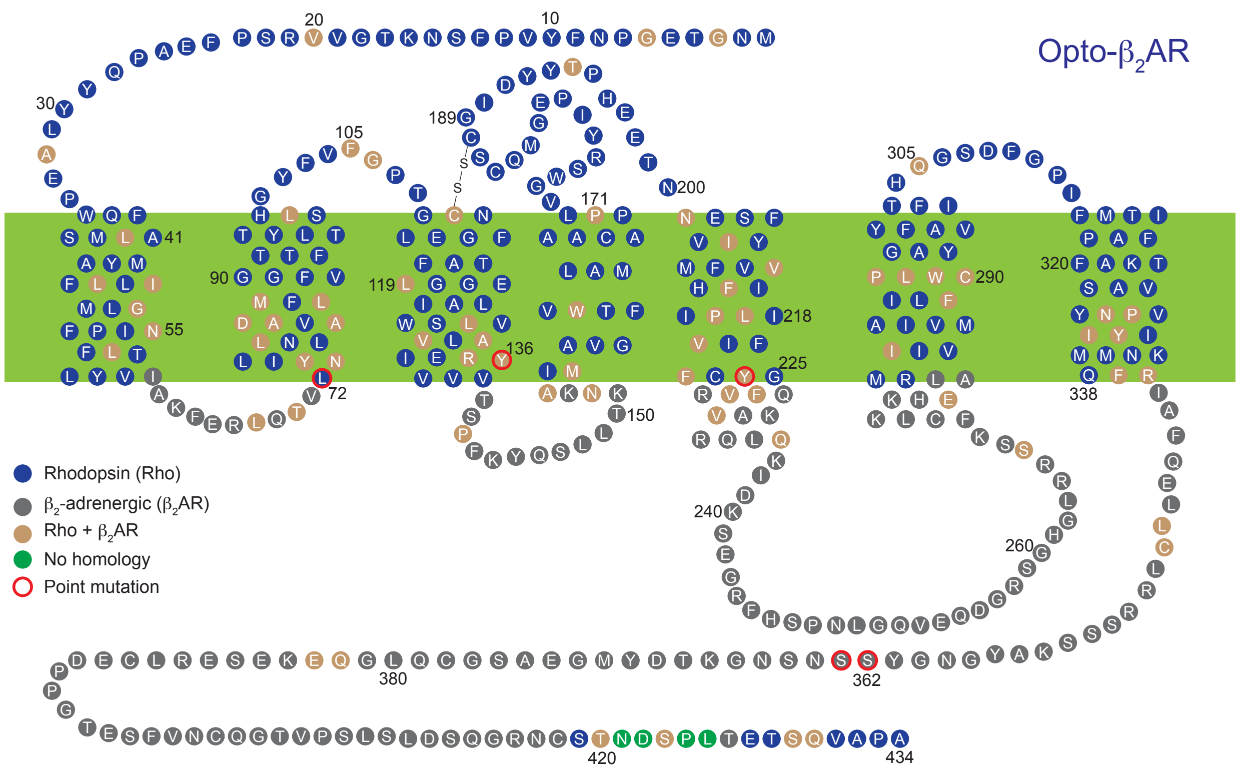

Supplementary Figure 4: Serpentine opto-β_2AR and mutant sequence. Cartoon sequence of opto-β_2AR depicting rhodopsin (blue) and β_2AR (brown) residues. Amino acids highlighted in red show residues mutated to arrestin-biased (opto-β_2AR^{L72F,Y136G,Y224A} or opto-β_2AR^{LYY}) and G-protein-biased (opto-β_2AR$^{S362A/S363G}$ or opto-β_2AR^{SS}) mutants. Similar mutations in arrestin-biased (β_2AR^{L72F,Y136G,Y224A} or β_2AR^{TYY}) and G-protein-biased (β_2AR^{S355A,S356G} or β_2AR^{SS}) β_2AR mutants.

Supplementary Figure 5

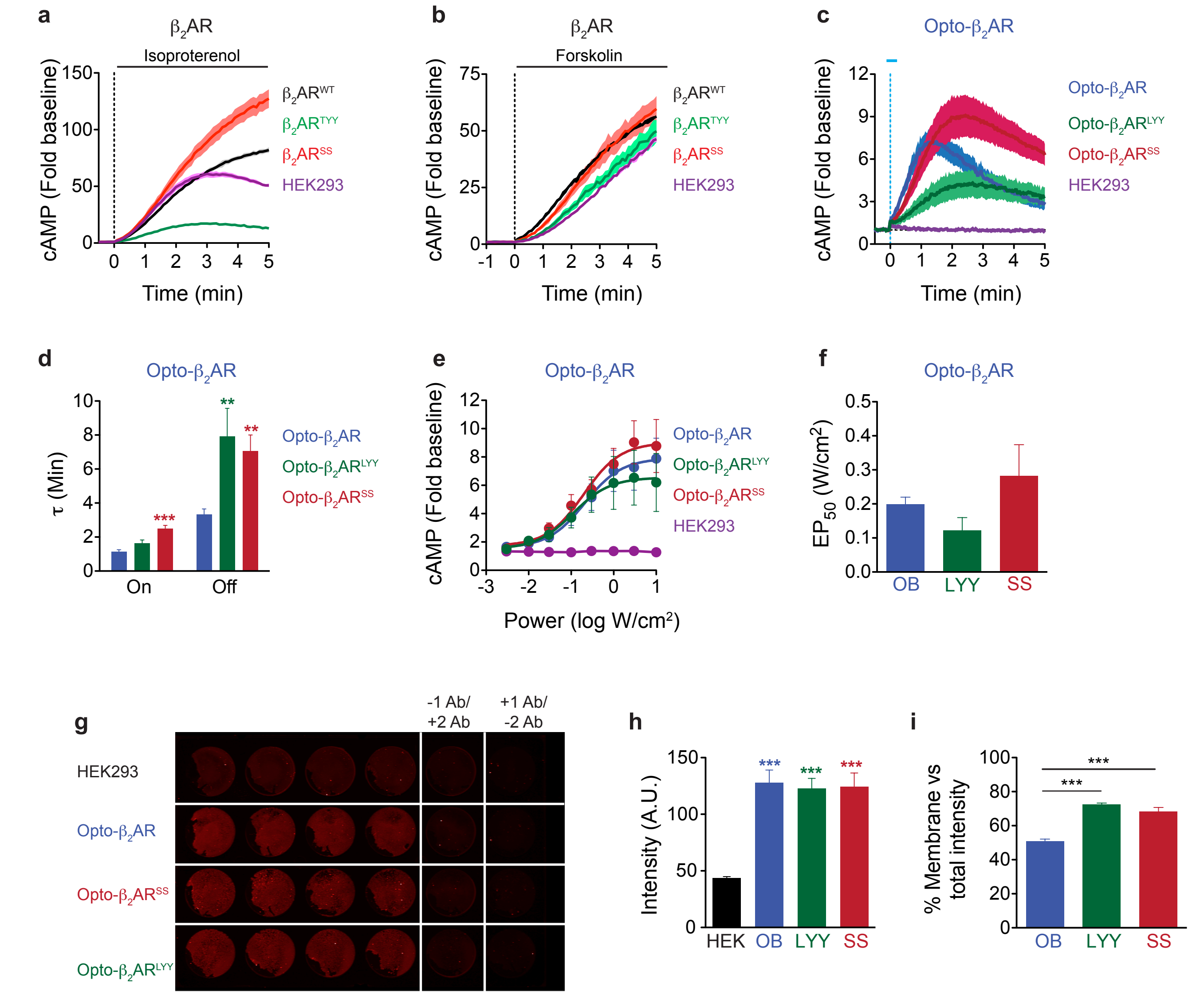

Supplementary Figure 5: Optical control of β-adrenergic signaling. (a) β_2AR^{WT} (black; n = 3), β_2AR^{TYY} (green; n = 3), β_2AR^{SS} (red; n = 3) and HEK293 (purple; n = 5) all activate cAMP in response to isoproterenol (1 μM) with differential kinetics. (**b**) β_2AR^{WT} (black; n = 3), β_2AR^{TYY} (green; n = 3), β_2AR^{SS} (red; n = 3) and HEK293 (purple; n = 2) all activate cAMP in response to forskolin (10 μM) with similar kinetic profiles. **(c)** Opto-β_2AR (blue; n = 15), opto-β_2AR^{LYY} (green; n = 8) and opto-β_2AR^{SS} (red; n = 17) all activate cAMP in response to a 5 sec blue light pulse. HEK293 transfected with just the GloSensor plasmid showed no effect to 5 sec light pulse. (**d**) Traces from (**c**) were fit with nonlinear one phase association (τ_{on}) and decay (τ_{off}) curves to obtain cAMP activation and deactivation time constants (**p< 0.01, ***p < 0.001 via One Way ANOVA followed by Dunnett's multiple comparison test to WT). (**e**) Opto-β_2AR (blue; n = 8), opto-β_2AR^{LYY} (green; n = 4) and opto-β_2AR^{SS} (red; n = 9) all activate cAMP in response to blue light in a power dependent manner. Untransfected HEK293 cells (purple; n = 4) containing the GloSensor plasmid show no response to any power of blue light. **(f)** Power response curves in **(e)** were fit with non-linear regression to produce EP_{50} values for opto-β_2AR (dark blue; n = 7), opto-β_2AR^{LYY} (dark green; n = 3) and opto-β_2AR^{SS} (dark red; n = 8). **(g)** Representative on cell western blot. **(h)** On cell western shows increased rhodopsin label in opto-β_2AR (dark blue; n = 4), opto-β_2AR^{LYY} (dark green; n = 4) and opto-β_2AR^{SS} (dark red; n = 4) over untransfected HEK293 cells alone (black; n = 4) (*** p < 0.001 via One Way ANOVA followed by Dunnett's multiple comparison test to HEK293 cells alone). **(i)** Percent of surface fluorescence to total fluorescence for opto-β_2AR (OB dark blue; n = 122), opto-β_2AR^{LYY} (LYY dark green; n = 55) and opto-β_2AR^{SS} (SS dark red; n = 15) (*** p < 0.001 via One Way ANOVA followed by Bonferroni's multiple comparison test. All data are expressed as mean ± SEM. All light pulses are 473 nm at 1W/cm^2 unless otherwise noted.

Supplementary Figure 6

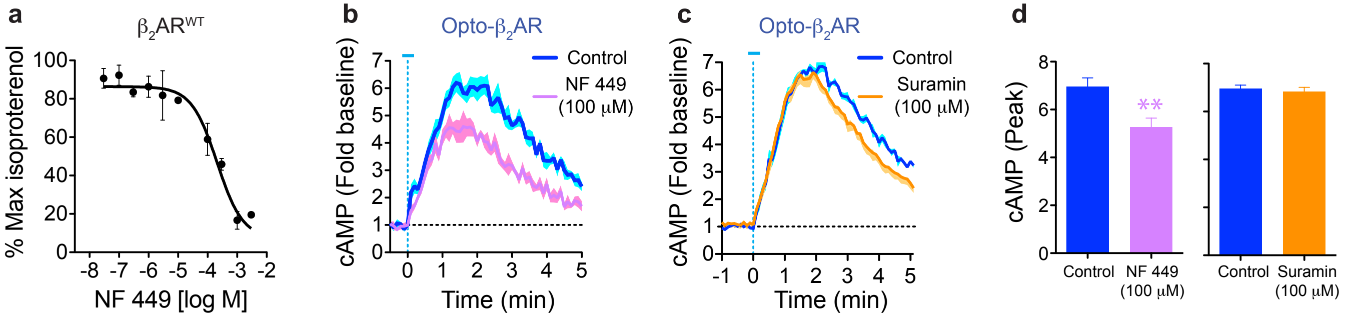

Supplementary Figure 6: Optical control of cAMP signaling. (a) NF449 shows a concentration dependent reduction in isoproterenol-induced (1 μM) cAMP in β_2AR^{WT} (black; n = 4) expressing cells (IC_{50} = 450 +/- 208 μM; n = 4). **(b)** Opto-β_2AR (n = 6 each treatment group) expressing cells show a reduction in light (5 sec)-induced cAMP in the presence of NF 449 (lilac; 100 μM). **(c)** Opto-β_2AR (n = 6 each treatment group) expressing cells show a reduction in light (5 sec)-induced cAMP in the presence of suramin (orange; 100 μM). **(d)** NF 449, but not suramin, shows a significant reduction in peak cAMP (** p = 0.0083 via Student's unpaired two-tailed t-test; n = 6 each group). All data are at room temperature unless otherwise noted. All data are expressed as mean ± SEM. All light pulses are 473 nm at 1W/cm^2 unless otherwise noted.

Supplementary Figure 7

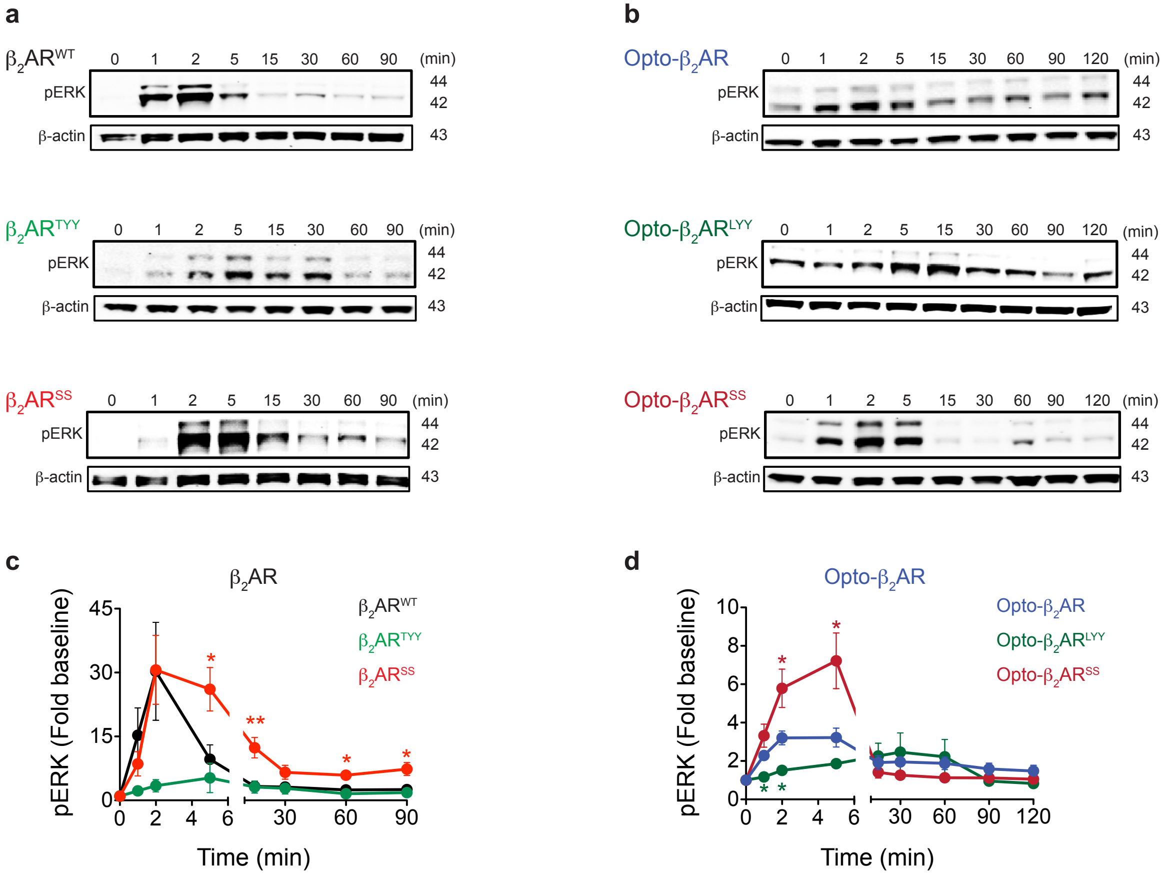

Supplementary Figure 7: Optical control of pERK signaling. (a) Representative immunoblots show pERK timecourse for β_2AR^{WT} (black), β_2AR^{TYY} (green) and β_2AR^{SS} (red). (**b**) Representative immunoblots show pERK timecourse for opto-β_2AR (dark blue), opto-β_2AR^{LYY} (dark green) and opto-β_2AR^{SS} (dark red). (**c**) Time course of isoproterenol (1 μM)-induced pERK expressed as fold baseline for β_2AR^{WT} (black; n = 5), β_2AR^{TYY} (green; n = 4) and β_2AR^{SS} (red; n = 3) (*p < 0.05, **p < 0.01 via Students' unpaired t-test to WT). (**d**) Time course of light (1 min)-induced pERK expressed as fold baseline for opto-β_2AR (dark blue; n = 10), opto-β_2AR^{LYY} (dark green; n = 4) and opto-β_2AR^{SS} (dark red; n = 7) (*p < 0.05 via Students' unpaired t-test to WT). All data are expressed as mean ± SEM. All light pulses are 473 nm at 1 W/cm^2 unless otherwise noted.

Supplementary Figure 8

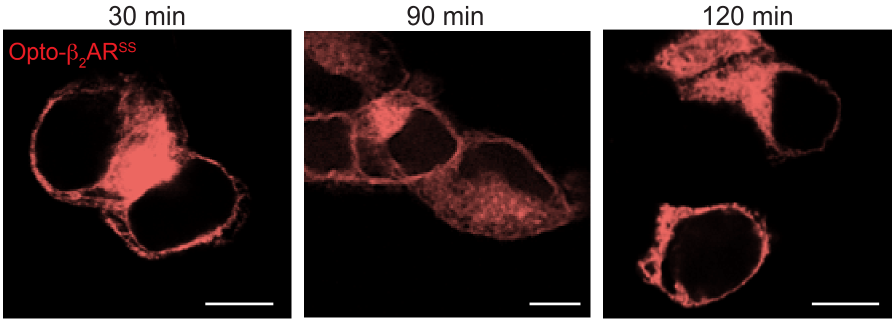

Supplementary Figure 8: Opto-β_2AR^{ss} internalization. Additional time points show lack of opto-β_2AR^{ss} (pseudocolored red) following photostimulation. Scale bar = 10 μm.

Supplementary Figure 9

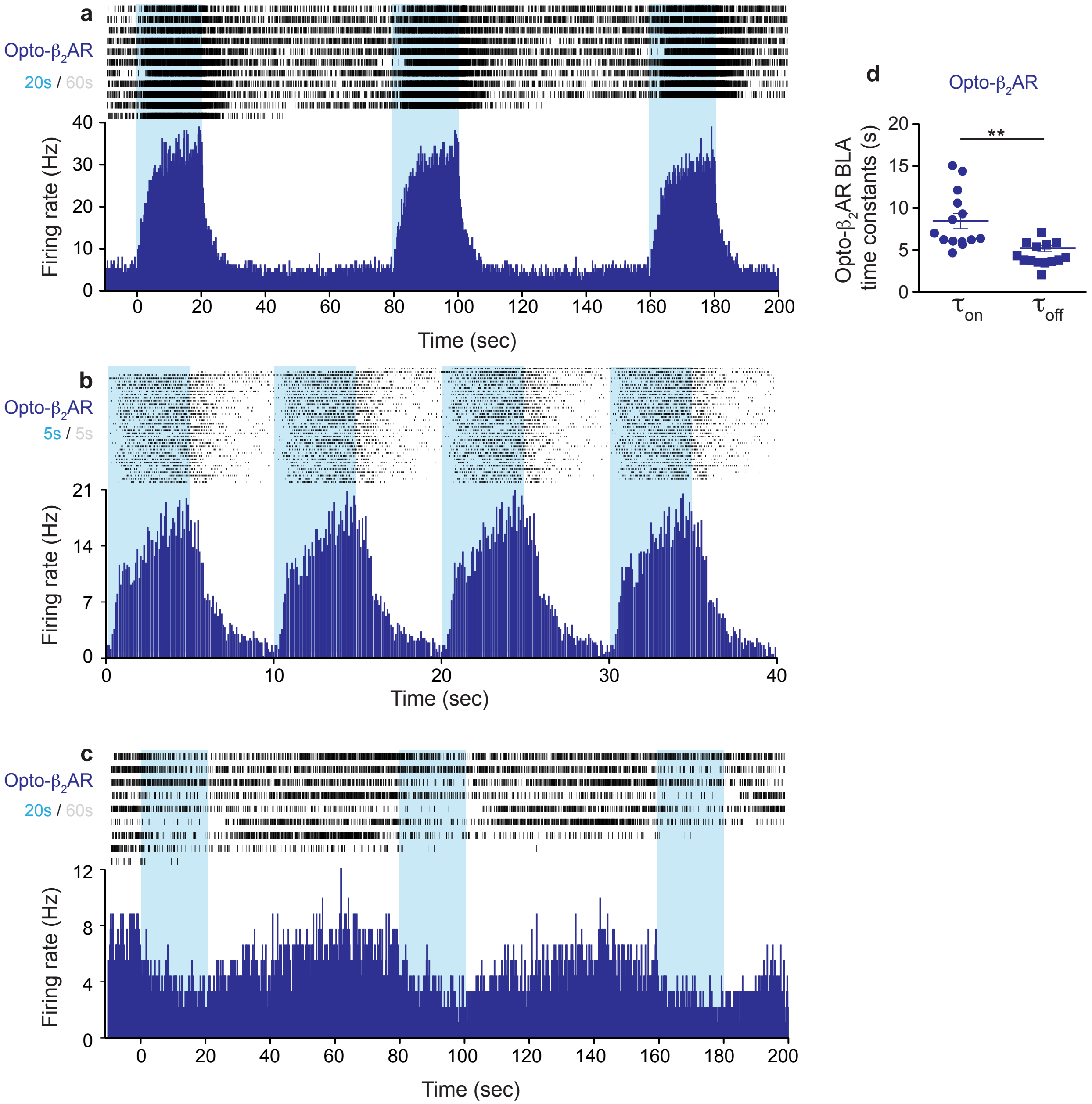

Supplementary Figure 9: Opto-β_2AR expressing CamKIIα BLA neurons are optically sensitive *in vivo* and drive excitatory activity. Representative histograms (100 ms bin) of isolated single unit showing *increase* in neuronal firing in opto-β_2AR expressing animals in response to: **(a)** 20 sec of light stimulation followed by 60 sec of no light, **(b)** 5 sec of light on, 5 sec of light off. **(c)** Representative histogram (100 ms bin) of isolated single unit shows *decrease* in neuronal firing in response to 20 sec light stimulation. **(d)** Neuronal responses to light (n = 12 units) in opto-β_2AR expressing animals fit with non-linear regression to obtain on (τ_{on}) and off (τ_{off}) time constants (**$p < 0.001$ via Students' unpaired t-test). All light pulses are 473 nm, 1 W/cm^2.

Supplementary Figure 10

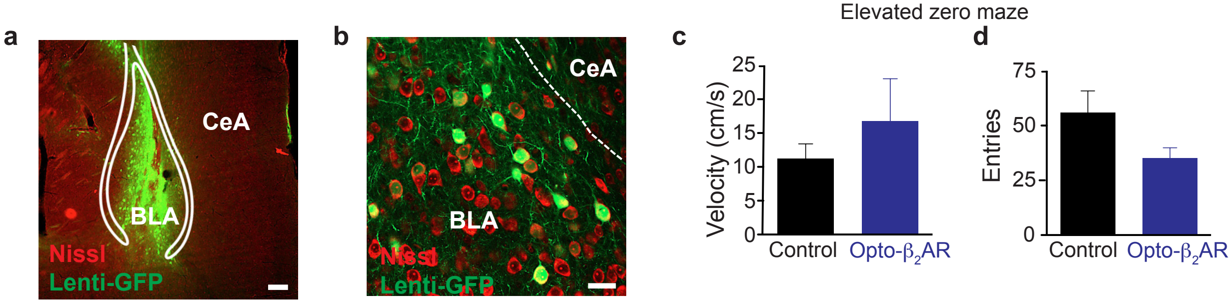

Supplementary Figure 10: Activation of Opto-β_2AR in the BLA promotes anxiety-like behavior. (**a - b**) Expression of lenti-EF1α-GFP (green) in the basolateral amygdala. Nissl pseudocolored red, scale bar = 100 μm (**a**) and 25 μm (**b**). (**c**) Average velocity and (**d**) average entries in EZM are not statistically different between opto-β_2AR (red, n = 7) and control animals (black; n = 10). All data expressed as mean ± SEM.

Supplementary Figure 11

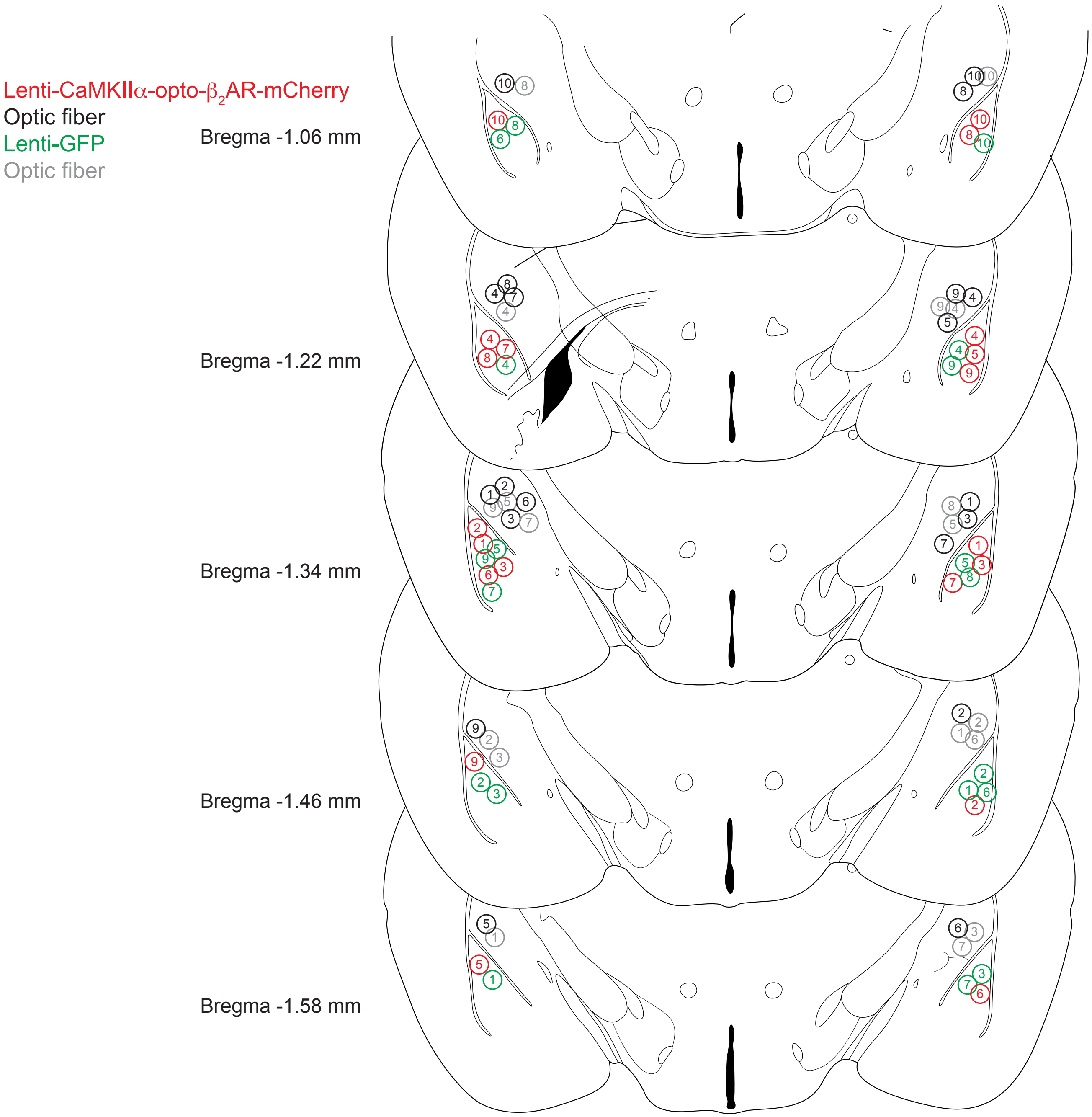

Supplementary Figure 11: Anatomical confirmation of viral expression and ferrule implants. Bilateral lenti-CaMKIIα-opto-β_2AR-mCherry (red) expression of 10 individual animals. Black is corresponding lenti-CaMKIIα-opto-β_2AR-mCherry fiber optic implant. Bilateral lenti-EF1α-GFP (green) expression of 10 individual animals. Grey is corresponding lenti-EF1α-GFP fiber optic implant. Numbers indicate individual animals and viral label was generally present throughout the intended target.

Supplementary Figure 12

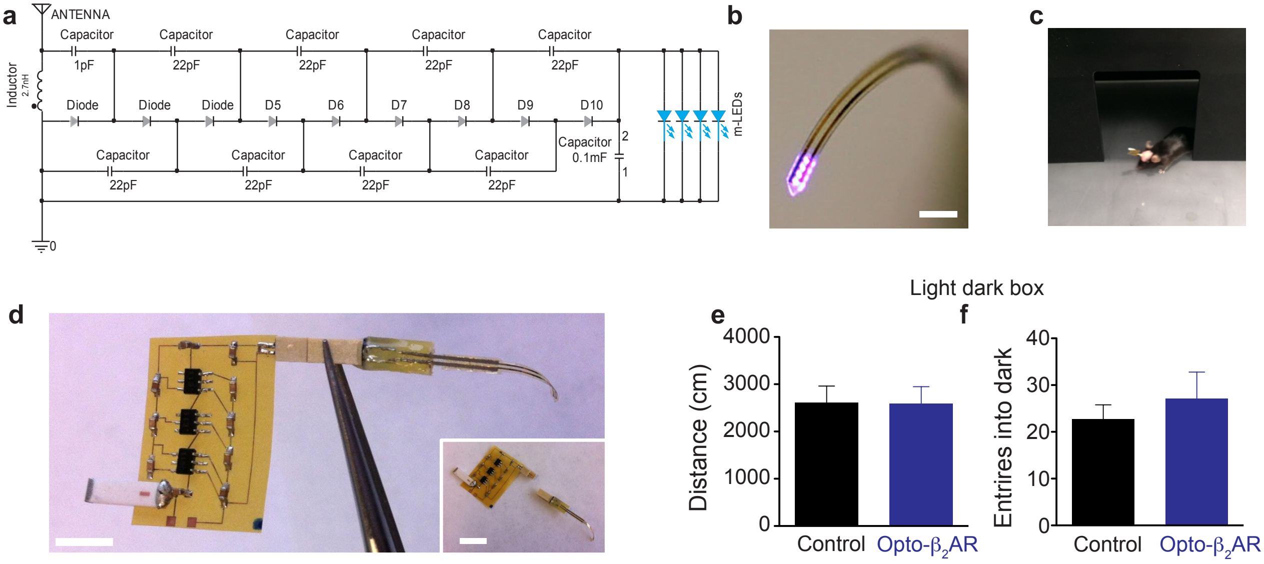

Supplementary Figure 12: Wireless photo-activation of Opto-β_2AR in the BLA promotes anxiety-like behavior. (**a**) Circuit diagram of an energy harvester operating at 1.5 Ghz. It consists of antenna, impedance matching circuits, Cockcroft-Walton multiplier, and micro-LEDs. (**b**) Enlarged view of wireless operation in the air. Scale bar = 1 mm. (**c**) Mouse with attached radiofrequency power harvester. (**d**) Radiofrequency power harvester with μ-LEDs. μ-LEDs are detachable from the harvester as shown in the inset. Scale bars = 5 mm. (**e**) Opto-β_2AR (red, n = 7) and control animals (black; n = 11) travel similar distances and make similar entries (**f**) into the dark in the light dark box assay. All data expressed as mean ± SEM.

Supplementary Figure 13

Figure 1f and Supplementary Figure 2a
β_2AR

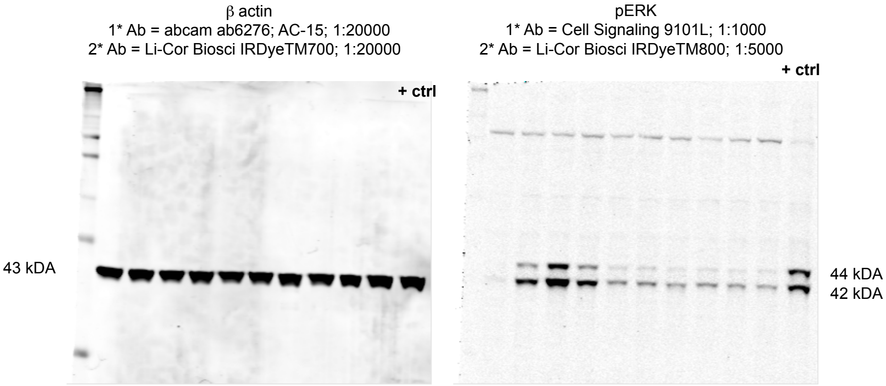

Figure 1f and Supplementary Figure 2a
Opto-β_2AR

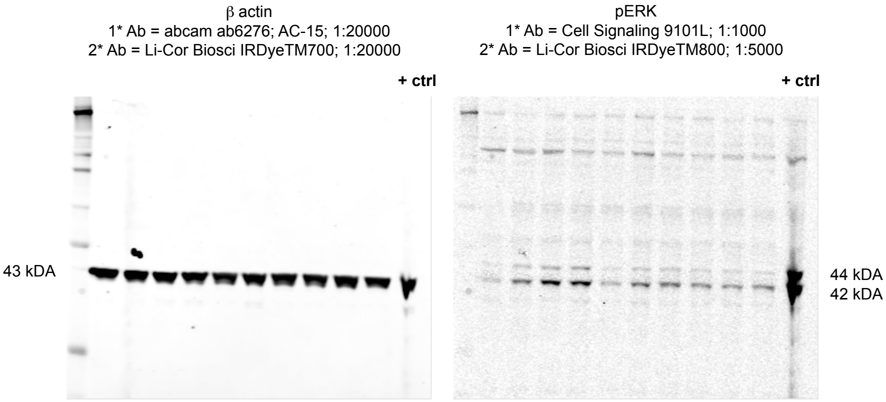

Supplementary Figure 2b
β_2AR (1min iso wash)

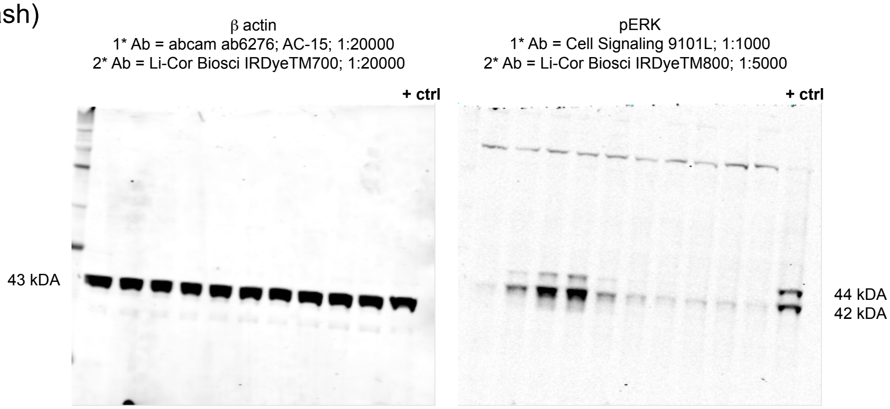

Supplementary Figure 13: Raw western blot gels.

Supplementary Figure 14

Supplementary Figure 2d
β_2AR (total ERK)

Total ERK
1* Ab = Cell Signaling (4696S); 1:7500
2* Ab = Li-Cor Biosci IRDyeTM700; 1:10000

pERK
1* Ab = Cell Signaling (9101L); 1:1000
2* Ab = Li-Cor Biosci nbbbbbbbbb IRDyeTM800; 1:5000

+ ctrl **+ ctrl**

44 kDA
42 kDA

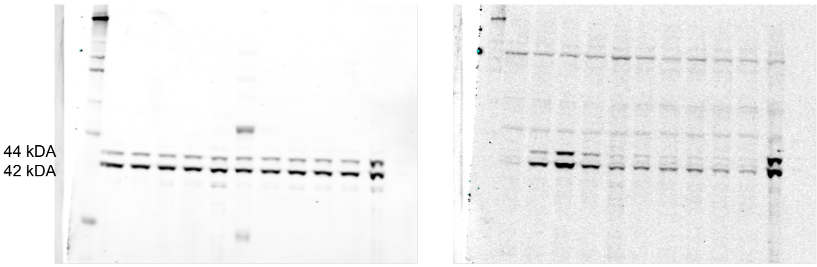

44 kDA
42 kDA

Supplementary Figure 2f
Opto-β_2AR (power)

β actin
1* Ab = abcam ab6276; AC-15; 1:20000
2* Ab = Li-Cor Biosci IRDyeTM700; 1:20000

pERK
1* Ab = Cell Signaling 9101L; 1:1000
2* Ab = Li-Cor Biosci IRDyeTM800; 1:5000

+ ctrl **+ ctrl**

43 kDA

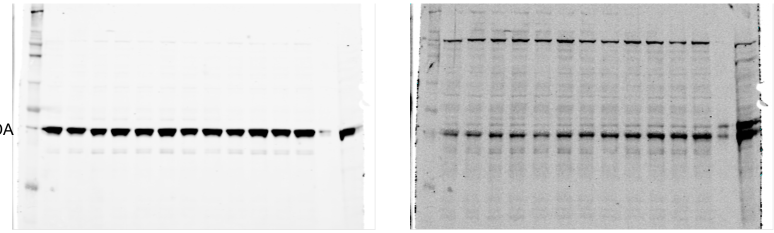

44 kDA
42 kDA

Supplementary Figure 2f
HEK293 (power)

β actin
1* Ab = abcam ab6276; AC-15; 1:20000
2* Ab = Li-Cor Biosci IRDyeTM700; 1:20000

pERK
1* Ab = Cell Signaling 9101L; 1:1000
2* Ab = Li-Cor Biosci IRDyeTM800; 1:5000

+ ctrl **+ ctrl**

43 kDA

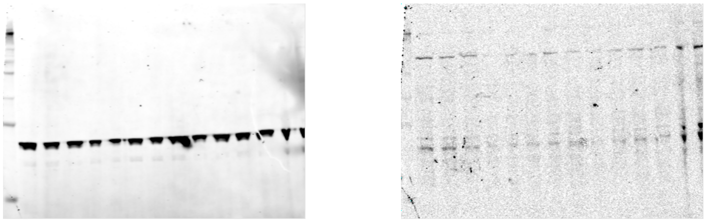

44 kDA
42 kDA

Supplementary Figure 2j
HEK293 and Opto-β_2AR
(dark activity)

β actin
1* Ab = abcam ab6276; AC-15; 1:20000
2* Ab = Li-Cor Biosci IRDyeTM700; 1:20000

pERK
1* Ab = Cell Signaling 9101L; 1:1000
2* Ab = Li-Cor Biosci IRDyeTM800; 1:5000

43 kDA

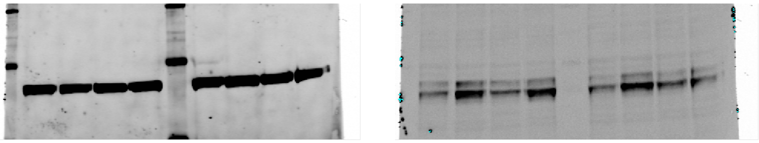

44 kDA
42 kDA

HEK293 Opto-β_2AR HEK293 Opto-β_2AR

Supplementary Figure 14: Raw western blot gels.

Supplementary Figure 15

Supplementary Figure 7a
β_2AR^{WT}

β actin
1* Ab = abcam ab6276; AC-15; 1:20000
2* Ab = Li-Cor Biosci IRDyeTM700; 1:20000
+ ctrl

43 kDA

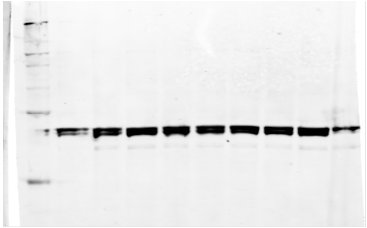

pERK
1* Ab = Cell Signaling 9101L; 1:1000
2* Ab = Li-Cor Biosci IRDyeTM800; 1:5000
+ ctrl

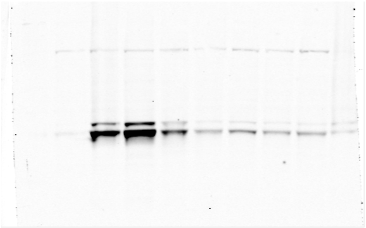

44 kDA
42 kDA

Supplementary Figure 7a
β_2AR^{TYY}

β actin
1* Ab = abcam ab6276; AC-15; 1:20000
2* Ab = Li-Cor Biosci IRDyeTM700; 1:20000
+ ctrl

43 kDA

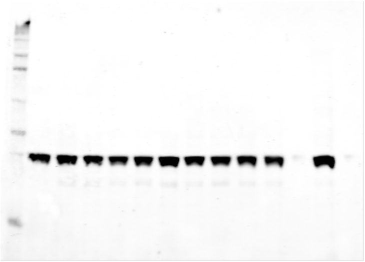

pERK
1* Ab = Cell Signaling 9101L; 1:1000
2* Ab = Li-Cor Biosci IRDyeTM800; 1:5000
+ ctrl

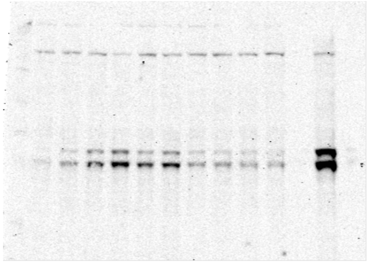

44 kDA
42 kDA

Supplementary Figure 7a
β_2AR^{SS}

β actin
1* Ab = abcam ab6276; AC-15; 1:20000
2* Ab = Li-Cor Biosci IRDyeTM700; 1:20000

43 kDA

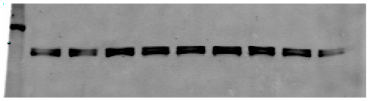

pERK
1* Ab = Cell Signaling 9101L; 1:1000
2* Ab = Li-Cor Biosci IRDyeTM800; 1:5000

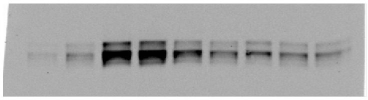

44 kDA
42 kDA

Supplementary Figure 15: Raw western blot gels.

Supplementary Figure 16

Supplementary Figure 7a
Opto-β_2AR^{WT}

β actin
1* Ab = abcam ab6276; AC-15; 1:20000
2* Ab = Li-Cor Biosci IRDyeTM700; 1:20000

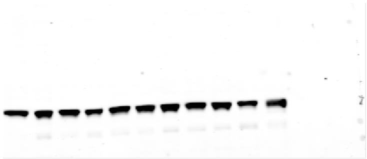

pERK
1* Ab = Cell Signaling 9101L; 1:1000
2* Ab = Li-Cor Biosci IRDyeTM800; 1:5000

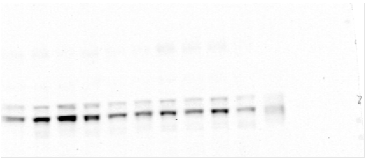

Supplementary Figure 7a
Opto-β_2AR^{TYY}

β actin
1* Ab = abcam ab6276; AC-15; 1:20000
2* Ab = Li-Cor Biosci IRDyeTM700; 1:20000

+ ctrl

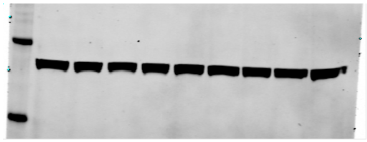

pERK
1* Ab = Cell Signaling 9101L; 1:1000
2* Ab = Li-Cor Biosci IRDyeTM800; 1:5000

+ ctrl

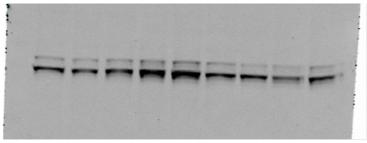

Supplementary Figure 7a
Opto-β_2AR^{SS}

β actin
1* Ab = abcam ab6276; AC-15; 1:20000
2* Ab = Li-Cor Biosci IRDyeTM700; 1:20000

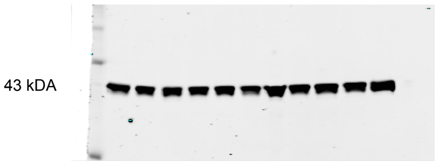

pERK
1* Ab = Cell Signaling 9101L; 1:1000
2* Ab = Li-Cor Biosci IRDyeTM800; 1:5000

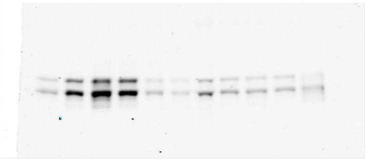

Supplementary Figure 16: Raw western blot gels.

Supplementary Table 1

	β_2AR	opto- β_2AR
**cAMP**		
EC_{50}/EP_{50}	14 ± 6 nM	0.9 ± 0.1 W/cm^2
τ_{on} (min)	0.76 ± 0.14	0.85 ± 0.03
τ_{off} (min)	0.89 ± 0.09	0.86 ± 0.1
**pERK**		
EP_{50}	1-3 μM[25]	4.3 ± 1.4 W/cm^2
**internalization**		
τ_{on} (min)	2.8	2.8
**recovery from desensitization**		
τ_{rec} (min)	11.2-16.7[34,59,60]	49
**neuronal activity**		
τ_{on} (sec)	-	8.1 ± 1
τ_{off} (sec)	-	4.8 ± 0.4

Supplementary Table 1 - summary of values obtained comparing β_2AR and opto-β_2AR. Select referenced values obtained from literature.

Supplementary Table 2

cAMP		opto- β_2AR	opto- β_2AR^{SS}	opto- β_2AR^{LYY}	β_2AR^{WT}	β_2AR^{SS}	β_2AR^{TYY}
EC_{50}/EP_{50}	25°C	0.2 ± 0.02	0.3 ± 0.09	0.12 ± 0.04	3.8 ± 0.4	1.4 ± 0.1	0.7 ± 0.2
τ_{on} (min)	37°C	0.46 ± 0.09	0.64 ± 0.09	-	0.9 ± 0.4	1.4 ± 0.1	0.7 ± 0.2
τ_{off} (min)	37°C	0.64 ± 0.09	2.2 ± 0.3	-	1.2 ± 0.01	5.5 ± 0.7	1.6 ± 0.1
τ_{on} (min)	25°C	1.1 ± 0.1	2.5 ± 0.2	1.6 ± 0.2	3.6 ± 0.5	4.1 ± 0.4	2.2 ± 0.2
τ_{off} (min)	25°C	3.3 ± 0.3	7 ± 1	7.9 ± 1.7	3.3 ± 0.04	5 ± 0.8	4.7 ± 0.5

internalization

τ_{on} (min)	37°C	2.8	-	2.8	-	-	-

recovery from desensitization

τ_{rec} (min)	37°C	49	n/a	-	-	-	-

Supplementary Table 2 - summary of values obtained comparing β_2AR^{WT}, β_2AR^{SS}, β_2AR^{TYY},and opto-β_2AR, opto-β_2AR^{SS}, opto-β_2AR^{LYY}.

Supplementary References

59. Pippig, S., Andexinger, S. & Lohse, M. J. Sequestration and recycling of beta 2-adrenergic receptors permit receptor resensitization. Mol. Pharmacol. 47, 666–676 (1995).

60. Barak, L. S., Menard, L., Ferguson, S. S. G., Colapietro, A.-M. & Caron, M. G. The Conserved Seven-Transmembrane Sequence NP(X)2,3Y of the G-Protein-Coupled Receptor Superfamily Regulates Multiple Properties of the .beta.2-Adrenergic Receptor. Biochemistry (Mosc.) 34, 15407–15414 (1995).
